# Humic Acid Alleviates Aluminum Toxicity in *Citrus grandis* (L.) Osbeck: Insight from Growth, Gas Exchange, and Related Physiological Parameters

**DOI:** 10.3390/plants15152370

**Published:** 2026-07-31

**Authors:** Qian Shen, Tian-Tian Xia, Liang-Yuan Tong, Bin-Bin Lan, Wei-Lin Huang, Ti Wu, Xin Ye, Ning-Wei Lai, Li-Song Chen

**Affiliations:** 1 College of Resources and Environment, Fujian Agriculture and Forestry University, Fuzhou 350002, China; shenqian202302@163.com (Q.S.); 15982655984@163.com (T.-T.X.); tongliangyuan2002@163.com (L.-Y.T.); lanbinbin2022@163.com (B.-B.L.); lainingwei@163.com (N.-W.L.); 2 College of Environmental and Biological Engineering, Putian University, Putian 351100, China; huangwl1821993@163.com

**Keywords:** aluminum stress, *Citrus grandis* (L.) Osbeck, humic acid, macronutrient, nutrient homeostasis, photosynthetic performance

## Abstract

Most *Citrus* spp. trees in China are cultivated in acidic soils with low soil organic matter and high Al^3+^. The mechanisms of humic acid (HA) to alleviate Al^3+^ stress in plants remain unclear. ‘Sour pummelo’ (*Citrus grandis* (L.) Osbeck) seedlings were exposed to 0.5 (HA0.5), 0.1 (HA0.1), or 0 (HA0) mM sodium humate and 1.2 (Al1.2) or 0 (Al0) mM AlCl_3_·6H_2_O for 128 days. Thereafter, the research examined biomass; Al and mineral nutrients; leaf photosynthetic performance; and leaf and root nonstructural carbohydrates, reactive oxygen species metabolism, and related physiological parameters. Al1.2 significantly reduced whole plant dry weight (DW), root DW, leaf CO_2_ assimilation (A_CO2_), and chlorophyll *a + b* concentration by 61%, 45%, 61%, and 35%, respectively, at HA0, but only 48%, 17%, 44%, and 11%, respectively, at HA0.5. Further analysis suggested that the addition of HA endowed *Citrus* with Al resilience by the following several aspects: (*a*) lessened tissue (leaf, stem, and root) concentrations of Al and enhanced capacity to maintain macronutrient (S, K, Mg, Ca, N, and P) homeostasis at Al1.2; (*b*) improved capacity to combat oxidative stress at Al1.2; and (*c*) enhanced A_CO2_ and growth at Al1.2. Further analysis indicated that HA-mediated alleviation of growth decline caused by Al1.2 involved (*a*) reduced ability to absorb Al and less root-to-shoot Al transport and (*b*) increased ability to maintain macronutrient homeostasis and to combat oxidative stress; and that HA-mediated alleviation of leaf chlorophyll and A_CO2_ decline and photosynthetic electron transport chain impairment involved less leaf Al concentration and improved leaf macronutrient homeostasis. To conclude, the addition of HA lowered roots’ ability to absorb Al and tissue Al concentration and subsequently mitigated Al-toxic impairment to root growth and function, thereby enhancing the ability of plants to maintain macronutrient homeostasis, and hence alleviating Al1.2-stimulated oxidative damage and inhibition of A_CO2_ and growth.

## 1. Introduction

Aluminum (Al) exists mainly as harmless precipitates in slightly acidic or neutral soils. However, Al may be released from clay minerals into soil solutions under acidic conditions, appearing as phytotoxic Al^3+^ [1]. Micromolar levels of Al^3+^ can rapidly inhibit root elongation and consequently disturb nutrient absorption, thereby leading to poor crop growth and yield loss [2,3]. Moreover, due to improper use of fertilizers and environmental pollution, the area of acidic soils is increasing instead of decreasing [4,5]. Additionally, Al^3+^ causes human health problems via the food chain [6]. Therefore, it is an urgent task to reveal the mechanisms of Al^3+^ toxicity and resilience in crops and develop agronomic measures to lower Al accumulation and alleviate Al^3+^ stress, which contributes to agricultural sustainability and ensures food safety.

In addition to disturbing nutrient absorption and inhibiting plant growth [7], Al^3+^ toxicity typically inhibits leaf photosynthesis in various plants, including *Citrus* spp. [8,9,10,11,12,13,14,15,16]. Simon et al. [12] indicated that Al^3+^-induced CO_2_ assimilation (A_CO2_) decline was at least partially brought about by stomatal closure, because Al^3+^ stress reduced both intercellular CO_2_ concentration (C_i_) and stomatal conductance (g_s_). However, growing evidence shows that nonstomatal factors are the main cause of photosynthesis decline, because Al^3+^-induced reductions in photosynthesis and g_s_ were accompanied by elevated or unchanged C_i_ and intercellular/ambient CO_2_ concentration ratios (C_i_/C_a_) [11,13,14,17]. The decline in leaf A_CO2_ caused by environmental perturbations such as Al^3+^ can be reflected by the behaviors of photosystem II (PSII), the most vulnerable constituent of the photosynthetic apparatus to unfavorable environmental conditions [17,18,19]. Previous studies indicated that the inhibition of A_CO2_ in leaves of Al-exposed *Citrus* leaves was associated with the damage of the whole photosynthetic electron transport chain (PETC) [20]. Peixoto et al. [15] attributed the reduction of photosynthesis in sorghum (*Sorghum bicolor* (L.) Moench) leaves to damaged PSII photochemistry, lessened pigment concentration, and possibly disturbed enzymatic machinery. However, the reduction of A_CO2_ in *Citrus* leaves induced by Al^3+^ was not mainly due to decreased chlorophyll (Chl) levels, because Al^3+^ toxicity reduced leaf A_CO2_ more than leaf Chl levels [21]. Simon et al. [12] suggested that Al^3+^ triggered a feedback inhibition of A_CO2_ in tomato leaves, because Al^3+^-induced root damage might decrease sucrose utilization and subsequently increase leaf carbohydrate accumulation. Unfortunately, the study did not examine root and leaf carbohydrates. Therefore, the mechanisms by which Al^3+^ stress causes a decline in A_CO2_ remain unclear.

Al^3+^ toxicity promotes reactive oxygen species (ROS) formation and accumulation in plant cells, thereby leading to lipid peroxidation [17,22]. Antioxidant enzymes play a crucial role in a plant’s resilience to Al^3+^ toxicity [23]. It was reported that Al^3+^-triggered increases in root malondialdehyde (MDA) and H_2_O_2_ concentrations were more pronounced in Al-sensitive maize (*Zea mays* L.) line than in an Al-resistant one, and that the antioxidant system was upregulated by Al^3+^ only in the Al-resistant line [22]. Similar findings have been reported on sorghum [24] and rice (*Oryza sativa* L.) [25]. Upregulation of antioxidant enzymes via the transgenic technique can endow plants with Al resistance [26,27].

Many *Citrus* orchards in China have acidic soils with low soil organic matter (SOM) levels [5], making *Citrus* trees susceptible to Al^3+^ toxicity and organic matter deficiency. Humic substances, composed of humic acid (HA), humin, and fulvic acid, are the primary component of SOM [28]. Humic substances are extremely diverse, deriving from various sources like brown coal, peat, mud, silt, and shale. The exact mechanisms by which humic substances influence plants remain unclear. Currently, research on the effects of humic substances on plants is mostly focused on HA and fulvic acid, especially the former [29,30,31,32,33,34,35]. Humic acid can not only improve soil health (physical stability, microbial diversity, and nutrient availability) [29,30] and nutrient acquisition [31,32], but also bind with metals via complexation (chelation), redox reactions, and adsorption to lower their mobility, bioavailability, and phytotoxicity [32,33]. Therefore, HA is regarded as an eco-friendly soil conditioner that can prevent metal toxicity in plants [34]. Evidence shows that in addition to reducing metal concentrations in plants [35], HA can enhance plant growth and relieve metal stress by promoting nutrient absorption, Chl biosynthesis [35,36], and photosynthesis [37,38] and lessening oxidative stress [35,36,39] under metal stress. So far, there are several studies showing that exogenous application of HA can attenuate plant Al^3+^ toxicity. It was reported that HA ameliorated maize root growth inhibition caused by Al^3+^ by complexing Al^3+^ and lowering monomeric Al (Al^3+^) in solution [40,41]. In maize seedlings, the addition of HA attenuated Al^3+^-induced decreases in leaf dry weight (DW) and P concentration and an increase in leaf Al concentration; whereas both HA and Al had little impact on leaf K concentration [42]. Notably, all of these studies used herbaceous plants as materials and focused on the impacts of HA on Al and nutrient uptake and plant growth under Al^3+^ toxicity. The mechanisms of HA to alleviate Al^3+^ stress in plants remain unclear.

Here, we examined the impacts of chemically synthesized HA supply on Al^3+^-triggered changes in growth; Al and nutrient absorption; leaf photosynthetic performance; and leaf and root nonstructural carbohydrates (NCs), total free amino acids (TFAAs), total soluble proteins (TSPs), and ROS metabolism in ‘Sour pummelo’ (*Citrus grandis* (L.) Osbeck) seedlings in order to ensure that the mitigation of Al^3+^ toxicity was mediated by HA rather than by other humic substances. The aim of the research was to corroborate the hypothesis that the addition of HA endowed *Citrus* with Al resistance through following several aspects: (*a*) lessened concentrations of tissue (leaf, stem, and root) Al and enhanced capacity to absorb nutrients and to maintain nutrient balance under Al^3+^ toxicity; (*b*) enhanced capacity to combat oxidative stress under Al^3+^ toxicity; and (*c*) increased growth and A_CO2_ under Al^3+^ stress.

## 2. Results

### 2.1. Addition of HA Alleviated Al-Triggered Decline of Biomass

**Figure 1:**
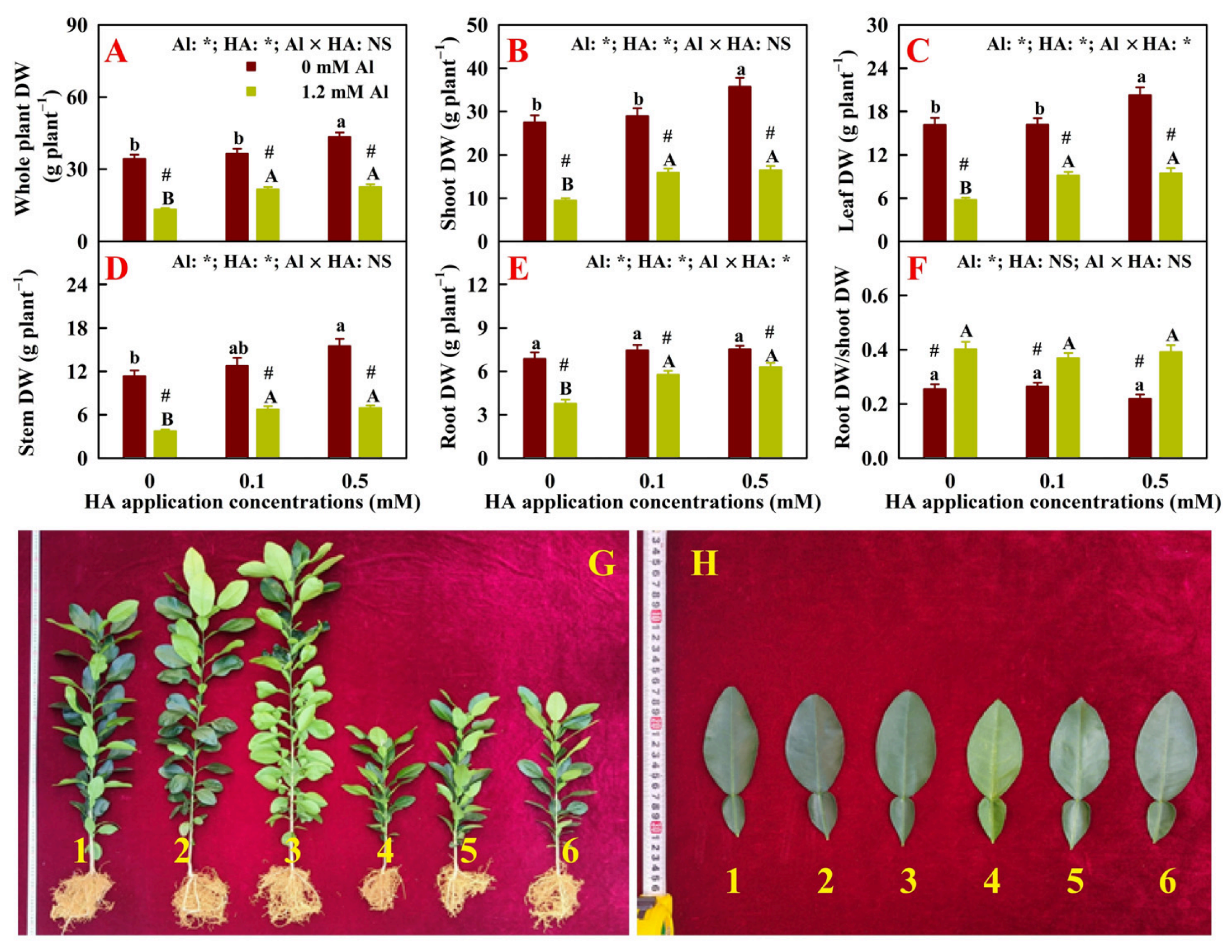
Impacts of Al-HA treatments on the mean (± SE, *n* = 10) whole plant dry weight (DW, (**A**)), shoot DW (**B**), leaf DW (**C**), stem DW (**D**), root DW (**E**), and root DW/shoot DW ratio (R/S, (**F**)), as well as growth (**G**,**H**) of ‘Sour pummelo’ seedlings. Bars with different lowercase and capital letters indicate significant differences at *p* ≤ 0.05 among three HA treatments within 0 and 1.2 mM Al, respectively. Bars with # indicate significant differences at *p* ≤ 0.05 between 0 and 1.2 mM Al treatments with the same HA treatment. NS, non-significant difference (*p* > 0.05); *, significant difference at *p* ≤ 0.05. HA, humic acid. 1, 0 mM Al (Al0) + 0 mM HA (HA0); 2, Al0 + 0.1 mM HA (HA0.1); 3, Al0 + 0.5 mM HA (HA0.5); 4, 1.2 mM Al (Al1.2) + HA0; 5, Al1.2 + HA0.1; 6, Al1.2 + HA0.5.

Aluminum stress (Al1.2) significantly lowered whole plant DW by 61%, 41%, and 48% (Figure 1A), shoot DW by 65%, 45%, and 54% (Figure 1B), leaf DW by 64%, 43%, and 53% (Figure 1C), stem DW by 67%, 47%, and 55% (Figure 1D), and root DW by 45%, 23%, and 17% (Figure 1E) at HA0, HA0.1, and HA0.5, respectively, but it augmented root DW/shoot DW ratio (R/S) by 59%, 40%, and 80% at HA0, HA0.1, and HA0.5, respectively (Figure 1F). Generally, biomass was augmented with the rise of HA supplementation at each given Al level (Figure 1A–E). The addition of HA did not significantly alter R/S at each given Al level (Figure 1F).

In addition to inhibiting plant growth, Al1.2 also caused leaf yellowing and necrosis, which was alleviated by HA supplementation (Figure 1G,H and Figure S1).

### 2.2. Alterations in the Concentrations, Uptake, and Distributions of Al and Nutrients Caused by Al-HA Treatments

**Figure 2:**
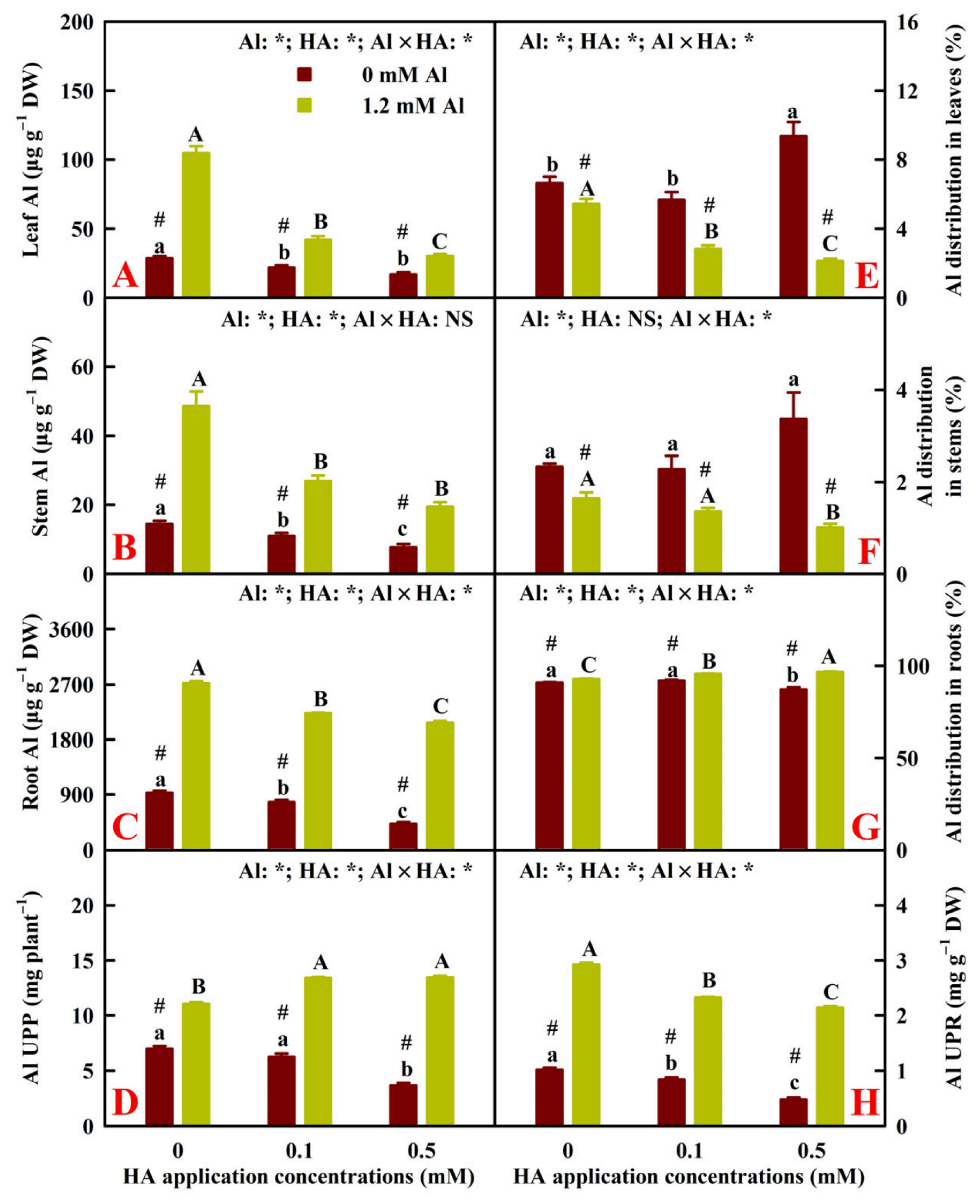
Impacts of Al-HA treatments on the mean (± SE, *n* = 4) Al concentrations in leaves (**A**), stems (**B**), and roots (**C**); Al uptake per plant (UPP, (**D**)); Al distributions in leaves (**E**), stems (**F**), and roots (**G**); and Al uptake per root dry weight (UPR, (**H**)) of ‘Sour pummelo’ seedlings. Bars with different lowercase and capital letters indicate significant differences at *p* ≤ 0.05 among three HA treatments within 0 and 1.2 mM Al, respectively. Bars with # indicate significant differences at *p* ≤ 0.05 between 0 and 1.2 mM Al treatments with the same HA treatment. NS, non-significant difference (*p* > 0.05); *, significant difference at *p* ≤ 0.05. HA, humic acid.

The research indicated that Al1.2 significantly augmented tissue (leaf, stem, and root) Al concentrations, Al distribution in roots, and Al uptake per root DW (UPR) and Al uptake per plant (UPP), but it significantly decreased Al distributions in leaves and stems. At Al1.2, leaf and root Al levels, Al UPR, and Al distributions in leaves lessened with the increase in HA supplementation; Al distribution in roots increased with the rise of HA supply; the addition of HA decreased and elevated stem Al concentration and Al UPP, respectively; and Al distribution in stems was higher at HA0 and HA0.1 than at HA0.5. At Al0, stem and root Al levels and Al UPR lessened with the increase in HA supplementation; leaf Al concentration was greater at HA0 than at HA0.1 and HA0.5; Al distribution in leaves was higher at HA0.5 than at HA0 and HA0.1; Al UPP and Al distribution in roots were higher at HA0 and HA0.1 than at HA0.5; and supply of HA had no significant influence on Al distribution in stems (Figure 2).

**Figure 3:**
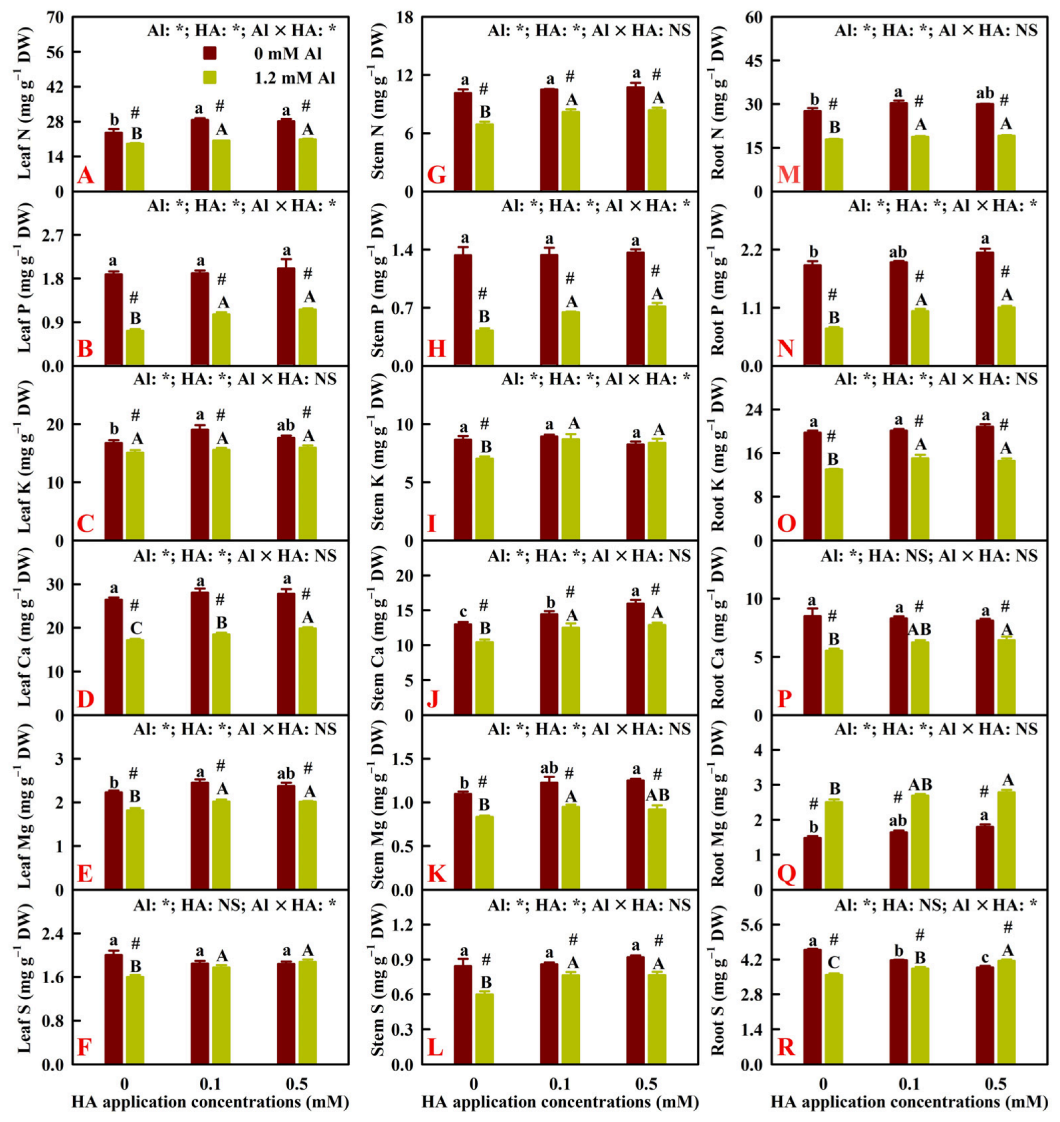
Impacts of Al-HA treatments on the mean (± SE, *n* = 4) concentrations of six macronutrients in leaves (**A**–**F**), stems (**G**–**L**), and roots (**M**–**R**). Bars with different lowercase and capital letters indicate significant differences at *p* ≤ 0.05 among three HA treatments within 0 and 1.2 mM Al, respectively. Bars with # indicate significant differences at *p* ≤ 0.05 between 0 and 1.2 mM Al treatments with the same HA treatment. NS, non-significant difference (*p* > 0.05); *, significant difference at *p* ≤ 0.05. HA, humic acid.

Figure 3 and Figure S2 exhibit the changes in nutrient concentrations in response to Al-HA treatments. Supplementation of HA prevented Al1.2-triggered reductions in tissue S, Ca, K, N, and P concentrations, except that the decreases in leaf and root N concentrations induced by Al1.2 were not greater at HA0 than at HA0.1 and HA0.5. Notably, Al1.2 significantly decreased leaf and stem Mg concentrations, but increased root Mg concentration. It was observed that addition of HA significantly increased or did not change tissue levels of macronutrients at each given Al level, except that at Al0, root S concentration decreased with the increase in HA supply (Figure 3).

The research showed that Al1.2 increased or did not alter tissue levels of micronutrients at each given HA level except that Al1.2 lessened the levels of Fe in leaves at HA0 and Cu in stems. At Al0, the addition of HA augmented or did not alter the levels of micronutrients in leaves, Cu, Mn, and Zn in stems, and Mn and Zn in roots, but it decreased or had no impact on the levels of Fe and B in stems and Cu, Fe, and B in roots. At Al1.2, the addition of HA increased or had no influence on the levels of Cu, Fe, and Zn in leaves, Cu, Mn, and Zn in stems, and Mn and Zn in roots, but it reduced or had no influence on the levels of Mn and B in leaves, Fe and B in stems, and Cu, Fe, and B in roots (Figure S2).

**Figure 4:**
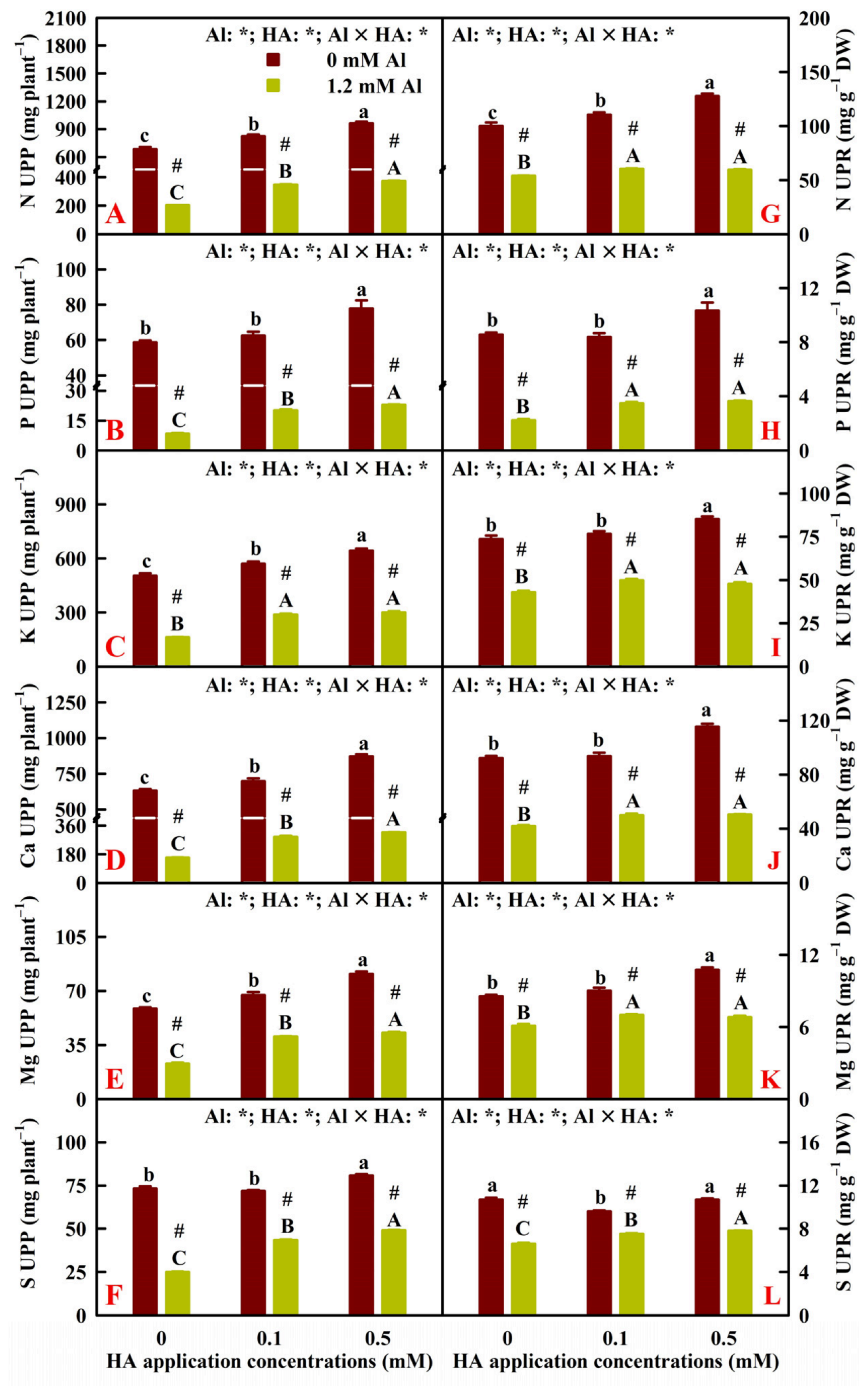
Impacts of Al-HA treatments on the mean (± SE, *n* = 4) N (**A,G**), P (**B**,**H**), K (**C**,**I**), Ca (**D**,**J**), Mg (**E**,**K**), and S (**F**,**L**) uptake per plant (UPP, (**A**–**F**)) and uptake per root dry weight (UPR, (**G**–**L**)). Bars with different lowercase and capital letters indicate significant differences at *p* ≤ 0.05 among three HA treatments within 0 and 1.2 mM Al, respectively. Bars with # indicate significant differences at *p* ≤ 0.05 between 0 and 1.2 mM Al treatments with the same HA treatment. *, significant difference at *p* ≤ 0.05. HA, humic acid.

Figure 4 and Figure S3 show the influence of Al-HA treatments on 11 nutrient UPP and UPR. Al1.2 significantly decreased macronutrient and B UPP and UPR and Cu and Fe UPP, but it increased Mn and Zn UPP and UPR and Cu and Fe UPR at each given HA level. The exceptions were that Al1.2 had no significant influence on Cu UPP at HA0.1, Fe UPP at HA0.1 and HA0.5, and B UPR at HA0 and HA0.1. The addition of HA increased or did not affect macronutrient, Zn, and Mn UPP and UPR, as well as Cu, Fe, and B UPP, but it decreased or did not affect Cu, Fe, and B UPR at each given Al level. The exception was that at Al0, S UPR was higher at HA0 and HA0.5 than at HA0.1.

**Figure 5:**
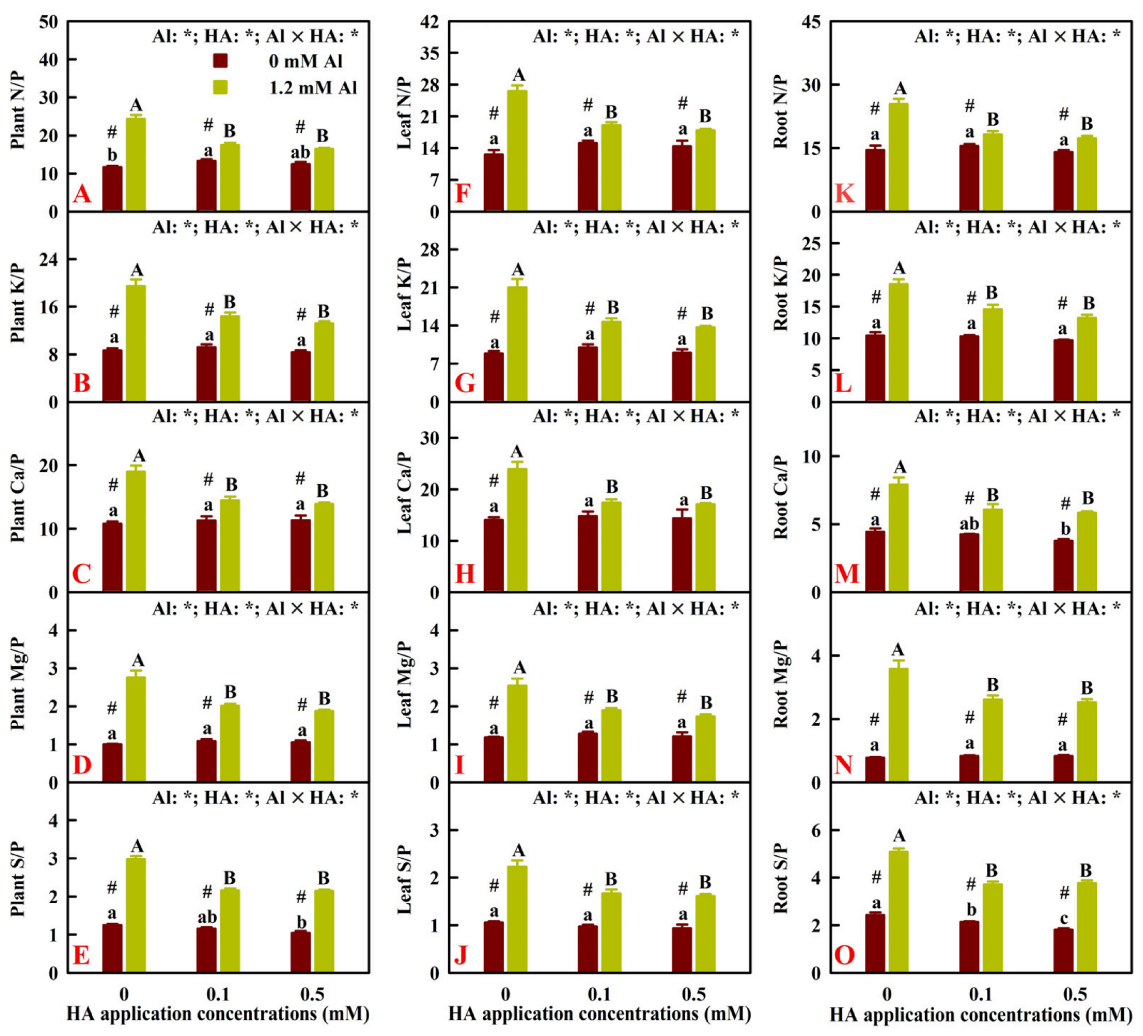
Impacts of Al-HA treatments on the mean (± SE, *n* = 4) ratios of N, K, Ca, Mg, and S uptake per plant (UPP) to P UPP (**A**–**E**), as well as ratios of N, K, Ca, Mg, and S concentrations to P concentration in leaves (**F**–**J**) and roots (**K**–**O**). Bars with different lowercase and capital letters indicate significant differences at *p* ≤ 0.05 among three HA treatments within 0 and 1.2 mM Al, respectively. Bars with # indicate significant differences at *p* ≤ 0.05 between 0 and 1.2 mM Al treatments with the same HA treatment. *, significant difference at *p* ≤ 0.05. HA: humic acid.

The study investigated the changes caused by Al-HA treatments in the ratios of S, Mg, N, K, and Ca UPP (UPR) to P UPP (UPR) (hereinafter referred to as plant (UPR) S/P, Mg/P, N/P, K/P, and Ca/P, respectively), as well as the ratios of S, Mg, N, K, and Ca concentrations to P concentration in leaves, roots, and stems, respectively (hereinafter referred to as leaf, root, and stem S/P, Mg/P, N/P, K/P, and Ca/P, respectively) (Figure 5 and Figure S4). The supplementation of HA alleviated Al1.2-stimulated rises in all these 25 ratios. At Al0, addition of HA did not alter any of these 25 ratios, with the exceptions that plant, root, and URP S/P and root Ca/P slightly decreased with the increase in HA supply, and that plant and UPR N/P were higher at HA0.1 than at HA0. At Al1.2, addition of HA reduced all these 25 ratios with the exception that stem K/P was not significantly lower at HA0.1 than at HA0.

**Figure 6:**
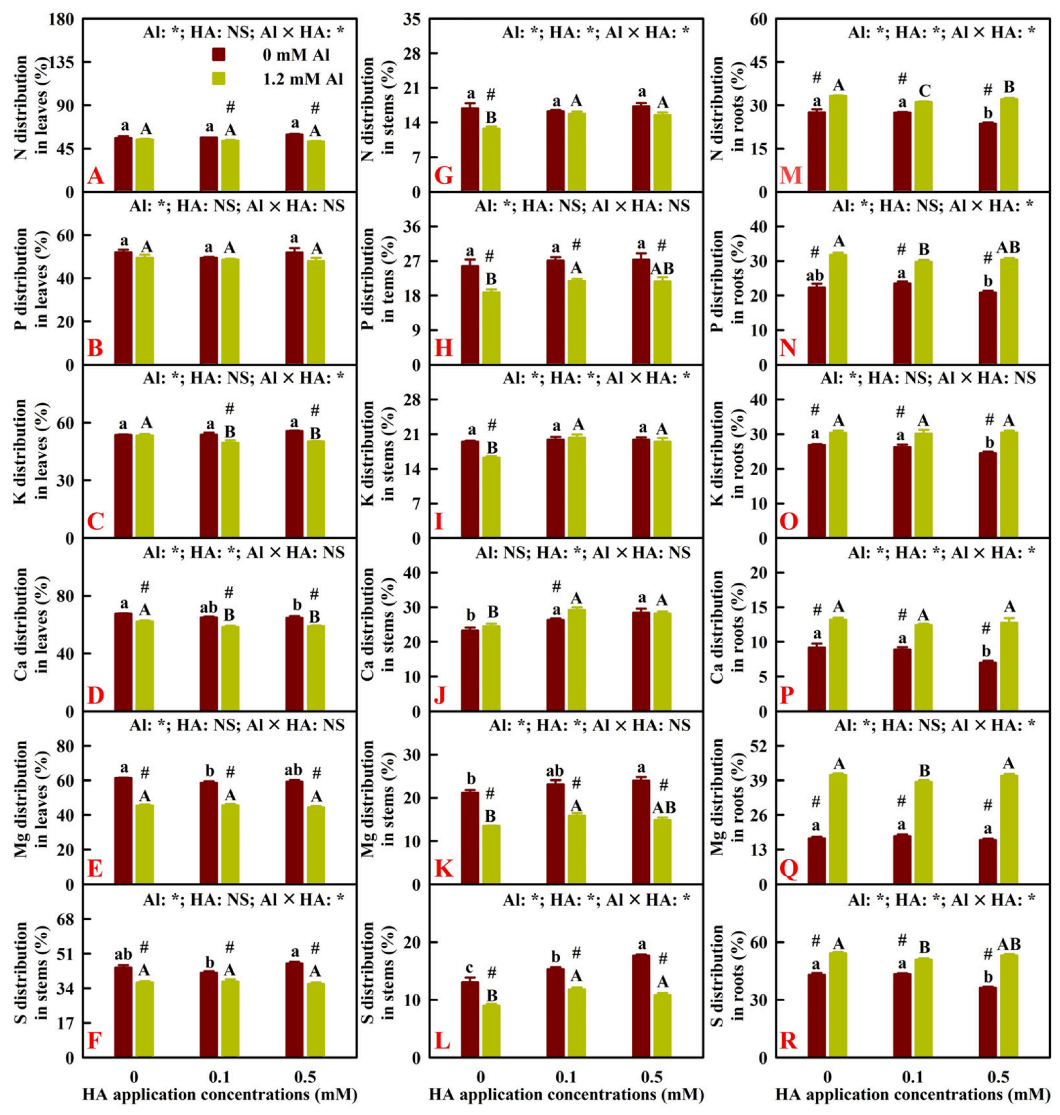
Impacts of Al-HA treatments on the mean (± SE, *n* = 4) distributions of N (**A**,**G**,**M**), P (**B**,**H**,**N**), K (**C**,**I**,**O**), Ca (**D**,**J**,**P**), Mg (**E**,**K**,**Q**), and S (**F**,**L**,**R**) in leaves (**A**–**F**), stems (**G**–**L**), and roots (**M**–**R**). Bars with different lowercase and capital letters indicate significant differences at *p* ≤ 0.05 among three HA treatments within 0 and 1.2 mM Al, respectively. Bars with # indicate significant differences at *p* ≤ 0.05 between 0 and 1.2 mM Al treatments with the same HA treatment. NS, non-significant difference (*p* > 0.05); *, significant difference at *p* ≤ 0.05. HA, humic acid.

Figure 6 and Figure S5 exhibit the changes caused by Al-HA treatments in the distributions of 11 nutrients in leaves, roots, and stems. Al1.2 lessened or did not change the distributions of all 11 nutrients in leaves and stems at each given HA level except for the distributions of Ca in stems at HA0.1 and B in stems at HA0.5, whereas Al1.2 elevated their distributions in roots. The addition of HA elevated or did not change the distributions of Cu, Fe, Mn, and B in leaves at Al0 and Cu, Zn, and Fe in leaves at Al1.2, but it decreased or did not change the distributions of Ca and Mg in leaves at Al0 and K, Ca, Mn, and B in leaves at Al1.2; the addition of HA enhanced or had no influence on the distributions of Ca, S, Mg, and Cu in stems at Al0 and macronutrient, Cu, Zn, and B in stems at Al1.2, but it lessened or had no impact on the distribution of Mn in stems at Al0; and the addition of HA lowered or did not change the distributions of S, Ca, K, N, Fe, Cu, and B in roots at Al0 and N, P, S, Mg, Fe, Cu, and Zn in roots at Al1.2, but the distribution of Mn in roots at Al1.2 was higher at HA0.1 and HA0.5 than at HA0. The addition of HA did not change the distribution of the other nutrients in leaves, stems, or roots at Al0 or Al1.2.

### 2.3. Addition of HA Reduced Al-Triggered Changes in Leaf Pigments, OJIP Transients, and Gas Exchange

**Figure 7:**
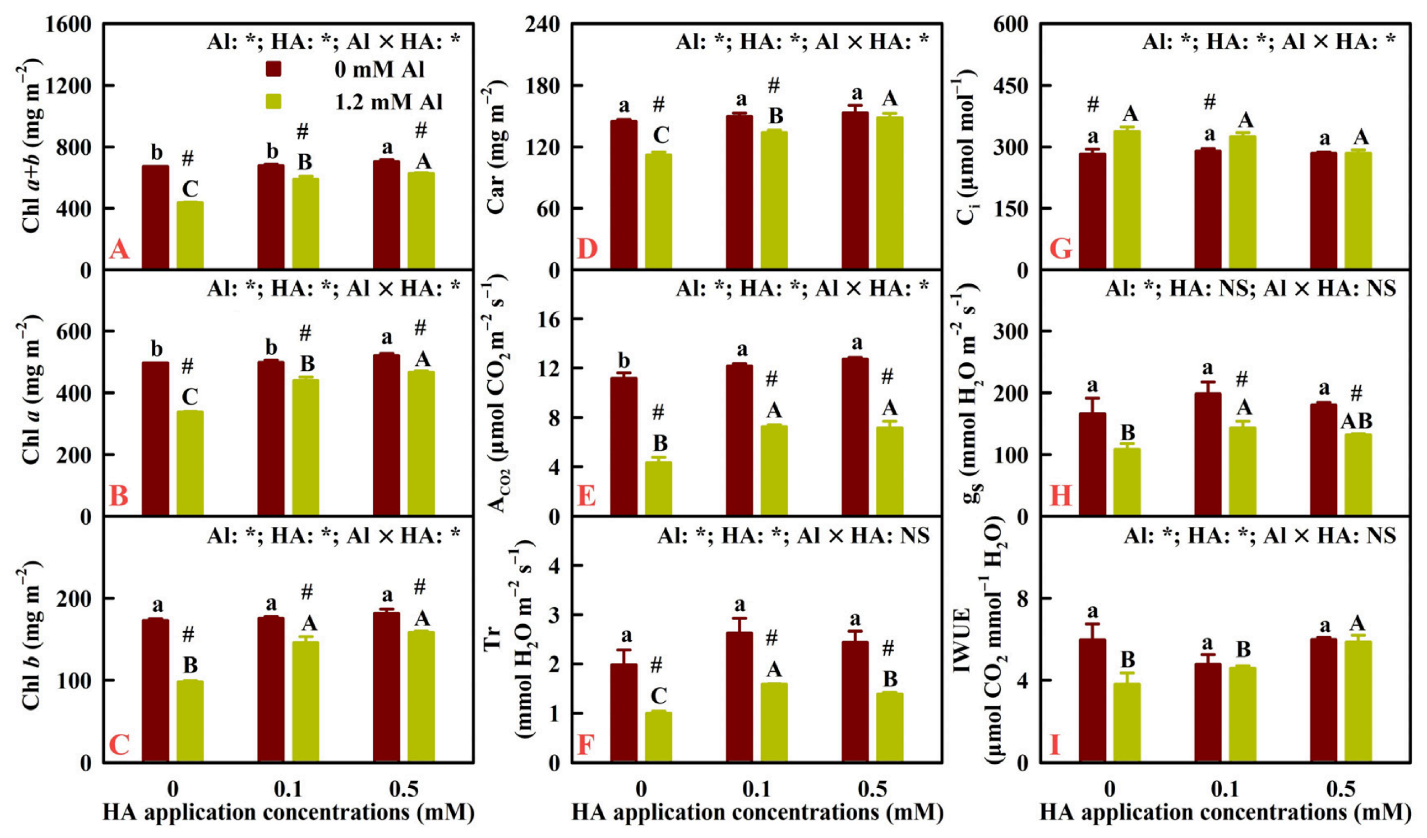
Impacts of Al-HA treatments on the mean (± SE, *n* = 4) Chl *a+b* (**A**), Chl *a* (**B**), Chl *b* (**C**), Car (**D**), A_CO2_ (**E**), T_r_ (**F**), C_i_ (**G**), g_s_ (**H**), and IWUE (**I**) in leaves. Bars with different lowercase and capital letters indicate significant differences at *p* ≤ 0.05 among three HA treatments within 0 and 1.2 mM Al, respectively. Bars with # indicate significant differences at *p* ≤ 0.05 between 0 and 1.2 mM Al treatments with the same HA treatment. NS, non-significant difference (*p* > 0.05); *, significant difference at *p* ≤ 0.05. A_CO2_, CO_2_ assimilation; Car, carotenoids; Chl, chlorophyll; C_i_, intercellular CO_2_ concentration; g_s,_ stomatal conductance; HA, humic acid; IWUE, instantaneous water use efficiency; T_r_, transpiration rate.

The addition of HA alleviated Al1.2-stimulated decreases in pigment levels. Their concentrations increased or did not change with the increasing supply of HA at each given Al level (Figure 7A–D).

The addition of HA ameliorated Al1.2-triggered decreases in A_CO2_ and T_r_ and an increase in C_i_ in leaves. Notably, Al1.2 significantly decreased g_s_ at HA0.1 and HA0.5, but not at HA0; Al1.2 had no significant influence on IWUE. At Al0, the addition of HA did not affect them, with the exception that A_CO2_ was greater at HA0.5 and HA0.1 than at HA0. At Al1.2, A_CO2_ and T_r_ were higher at HA0.1 and HA0.5 than at HA0; the addition of HA increased or did not alter g_s_ and IWUE; and HA supplementation had no significant impact on C_i_ (Figure 7E–I).

**Figure 8:**
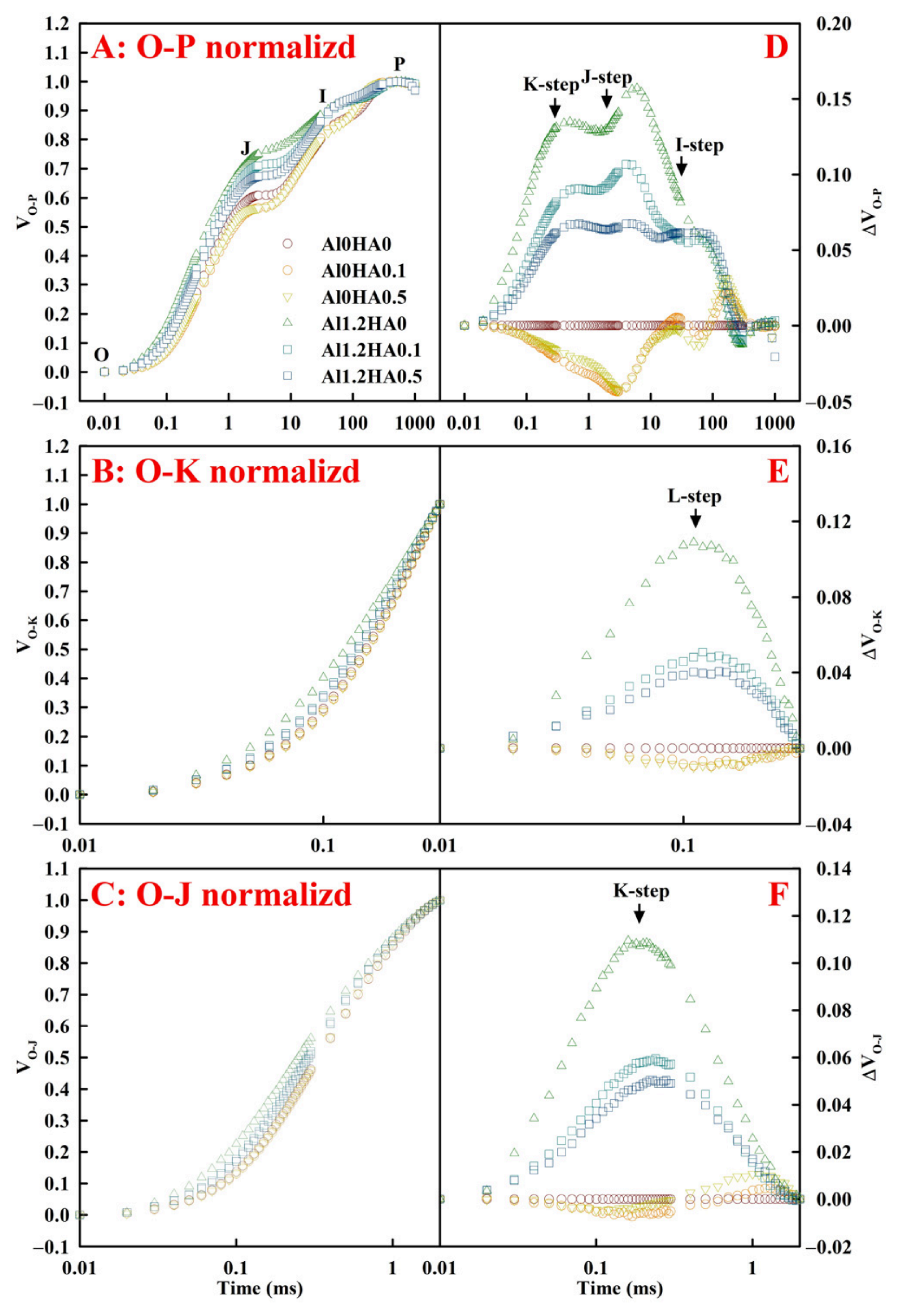
Impacts of Al-HA treatments on the mean (*n* = 10) OJIP transients expressed as the kinetics of relative variable fluorescence: between F_o_ and F_m_: V_O-P_ = (F_t_–F_o_)/(F_m_–F_o_) (**A**) and the differences of the six samples to the reference sample exposed to Al0HA0 (ΔV_O-P_, (**D**)); between F_o_ and F_300μs_: V_O-K_ = (F_t_–F_o_)/(F_300μ_–F_o_) (**B**) and the differences of the six samples to the reference sample (ΔV_O-K_, (**E**)); and between F_o_ and F_J_: V_O-J_ = (F_t_–F_o_)/(FJ–F_o_) (**C**) and the differences of the six samples to the reference sample (ΔV_O-J_, (**F**)). Al0HA0, 0 mM Al (Al0) + 0 mM HA (HA0); Al0HA0.1, Al0 + 0.1 mM HA (HA0.1); Al0HA0.5, Al0 + 0.5 mM HA (HA0.5); Al1.2HA0, 1.2 mM Al (Al1.2) + HA0; Al1.2HA0.1, Al1.2 + HA0.1; Al1.2HA0.5, Al1.2 + HA0.5; F_300μs_, fluorescence intensity at 300 μs; F_J_, fluorescence intensity at the J-step (2 ms); F_m_, maximum fluorescence; F_o_, minimum fluorescence; F_t_, fluorescence at time *t* after onset of actinic illumination; HA, humic acid.

It was observed that the OJIP transients from the leaves of Al1.2-exposed seedlings (LAl1.2) exhibited a positive ΔK-step at 300 μs, ΔJ-step at 2 ms, ΔI-step at 30 ms, and ΔL-step at ~130 μs compared with the OJIP transients from leaves of Al0HA0-exposed seedlings (LAl0HA0), and that the changes in OJIP transients caused by Al1.2 were lessened by augmenting HA supply. Notably, the OJIP transients from the leaves of Al0HA0.1- and Al0HA0.5-exposed seedlings had negative ΔK-, ΔJ-, and ΔL-steps compared with the OJIP transients from LAl0HA0 (Figure 8).

**Figure 9:**
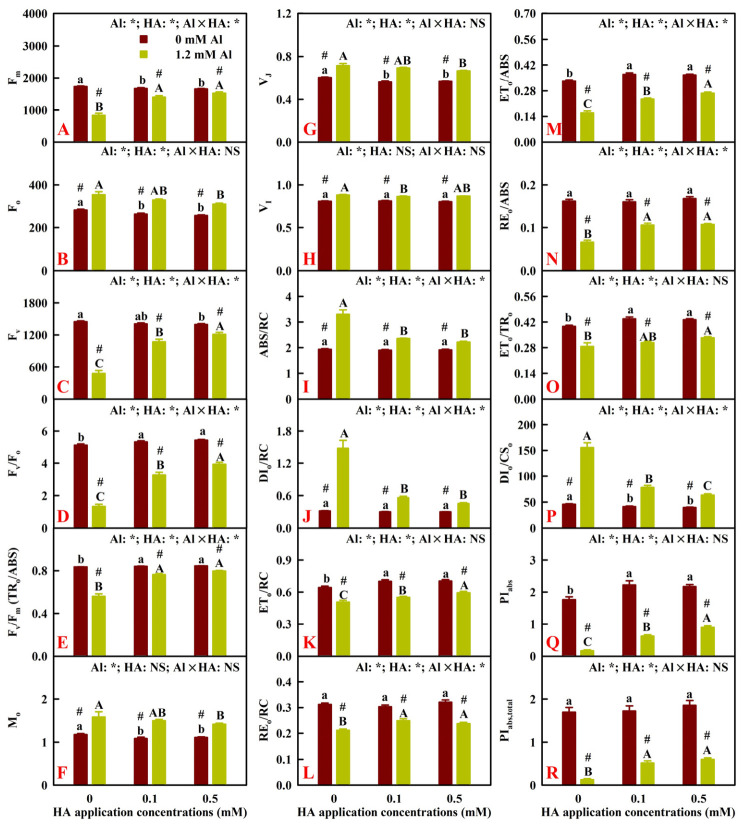
Impacts of Al-HA treatments on the mean values (±SE, *n* = 10) of F_m_ (**A**), F_o_ (**B**), F_v_ (**C**), F_v_*/*F_o_ (**D**), F_v_*/*F_m_ (TR_o_/ABS, (**E**)), M_o_ (**F**), V_J_ (**G**), V_I_ (**H**), ABS/RC (**I**), DI_o_/RC (**J**), ET_o_/RC (**K**), RE_o_/RC (**L**), ET_o_/ABS (**M**), RE_o_/ABS (**N**), ET_o_/TR_o_ (**O**), DI_o_/CS_o_ (**P**), PI_abs_ (**Q**), and PI_abs,total_ (**R**) in leaves. Bars with different lowercase and capital letters indicate significant differences at *p* ≤ 0.05 among three HA treatments within 0 and 1.2 mM Al, respectively. Bars with # indicate significant differences at *p* ≤ 0.05 between 0 and 1.2 mM Al treatments with the same HA treatment. NS, non-significant difference (*p* > 0.05); *, significant difference at *p* ≤ 0.05. ABS/RC, absorption flux per reaction center (RC) at *t* = 0; DI_o_/CS_o_, dissipated energy flux per cross section (CS) at *t* = 0; DI_o_/RC, dissipated energy flux per RC at *t* = 0; ET_o_/ABS, quantum yield for electron transport at *t* = 0; ET_o_/RC, electron transport flux per RC at *t* = 0; ET_o_/TR_o_, probability (at time 0) that a trapped exciton moves an electron into the electron transport chain beyond Q_A_^−^; F_m_, maximum fluorescence; F_o_, minimum fluorescence; F_V_, maximum variable fluorescence; F_v_/F_o_, maximum primary yield of photochemistry of photosystem II; HA, humic acid; M_o_, approximated initial slope (in ms^−1^) of the fluorescence transient *V* = *f(t)*; PI_abs_, performance index; PI_abs,total_, total performance index; RE_o_/ABS, quantum yield for the reduction of end acceptors of photosystem I per photon absorbed at *t* = 0; RE_o_/RC, reduction of end acceptors at photosystem I (PSI) electron acceptor side per RC at *t* = 0; TR_o_/ABS or F_v_/F_m_, maximum quantum yield of primary photochemistry; V_I_, relative variable fluorescence at the I-step (30 ms); V_J_, relative variable fluorescence at the J-step (2 ms).

It was found that the addition of HA alleviated Al1.2-stimulated rises in V_I_, V_J_, F_o_, M_o_, ABS/RC, DI_o_/RC, and DI_o_/CS_o_, and reductions in the other 11 fluorescence indexes. At Al1.2, supplementation of HA decreased or did not affect F_o_, M_o_, V_J_, V_I_, ABS/RC, DI_o_/RC, and DI_o_/CS_o_, and increased the other 11 fluorescence indexes, except that ET_o_/TR_o_ was not higher at HA0.1 than at HA0. At Al0, F_v_ significantly decreased with increasing HA supplementation; F_o_, F_m_, M_o_, V_J_, and DI_o_/CS_o_ were higher at HA0 than at HA0.1 and HA0.5; F_v_/F_o_, F_v_/F_m_, ET_o_/RC, ET_o_/ABS, ET_o_/TR_o_, and PI_abs_ were greater at HA0.1 and HA0.5 than at HA0; and the other six fluorescence indexes remained unchanged in response to HA supplementation (Figure 9).

### 2.4. Addition of HA Alleviated Al-Triggered Alterations in Leaf and Root Levels of NCs, TSPs, and TFAAs

**Figure 10:**
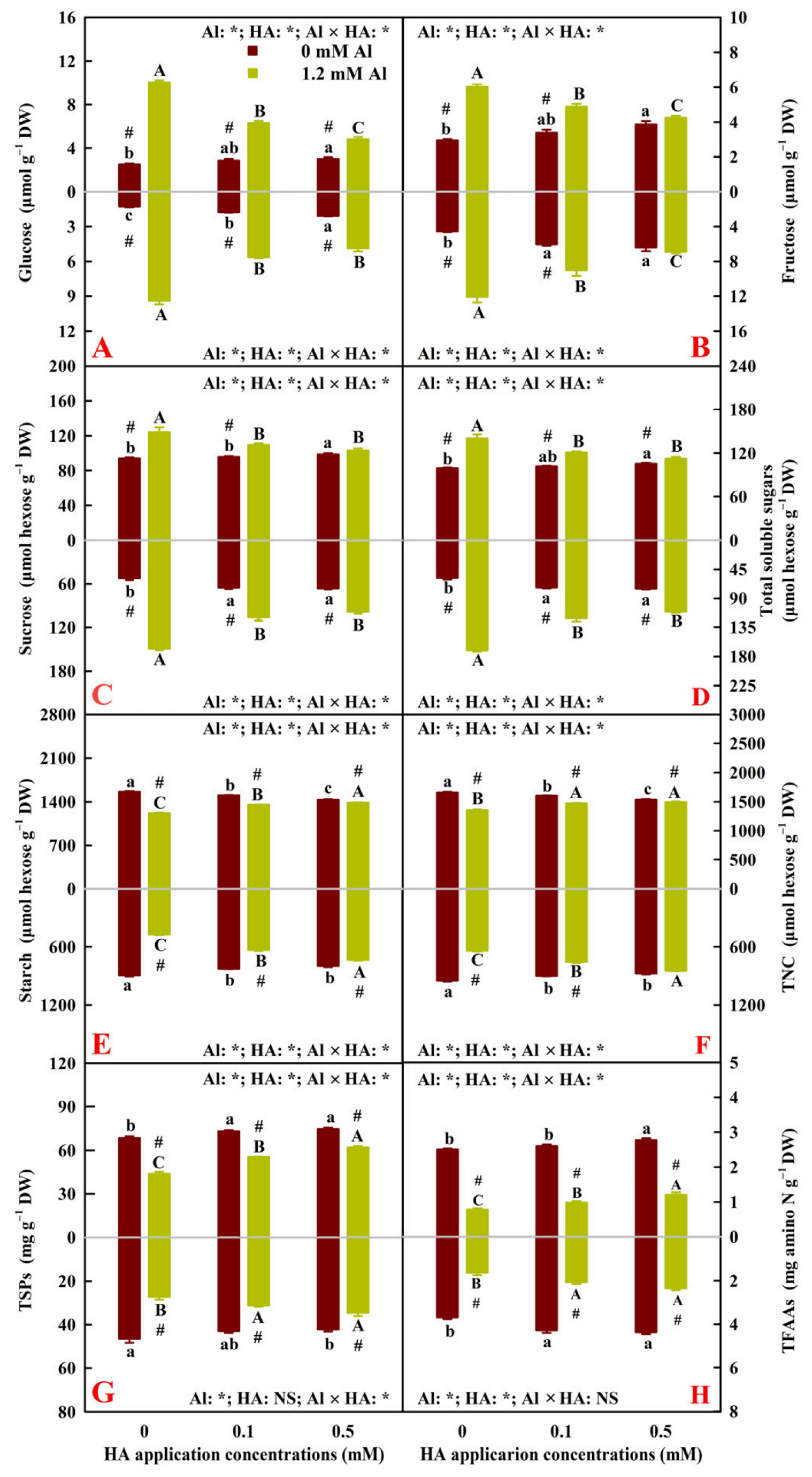
Impacts of Al-HA treatments on the mean (± SE, *n* = 4) concentrations of glucose (**A**), fructose (**B**), sucrose (**C**), total soluble sugars (glucose + fructose + sucrose, (**D**)), starch (**E**), and TNC (total soluble sugars + starch, (**F**)), TSPs (**G**), and TFAAs (**H**) in leaves (above column) and roots (below column). Bars with different lowercase and capital letters indicate significant differences at *p* ≤ 0.05 among three HA treatments within 0 and 1.2 mM Al, respectively. Bars with # indicate significant differences at *p* ≤ 0.05 between 0 and 1.2 mM Al treatments with the same HA treatment. NS, non-significant difference (*p* > 0.05); *, significant difference at *p* ≤ 0.05. HA, humic acid; TFAAs, total free amino acids; TNC, total nonstructural carbohydrates; TSPs, total soluble proteins.

The addition of HA prevented Al1.2-stimulated rises in leaf and root accumulation of soluble sugars and declines in leaf and root accumulation of starch, TNC, TSPs, and TFAAs. At Al1.2, the addition of HA decreased leaf and root accumulation of soluble sugars, but increased leaf and root accumulation of starch, TNC, TSPs, and TFAAs. At Al0, the addition of HA slightly increased or did not change leaf and root concentrations of soluble sugars, TSPs, and TFAAs, except that the root concentration of TSPs was higher at HA0 than at HA0.5, but it slightly decreased root and leaf levels of starch and TNC (Figure 10).

### 2.5. Addition of HA Attenuated Al-Triggered Increases in HPR and MDA Levels and Decreases in Antioxidant Enzyme Activities of Leaves and Roots

**Figure 11:**
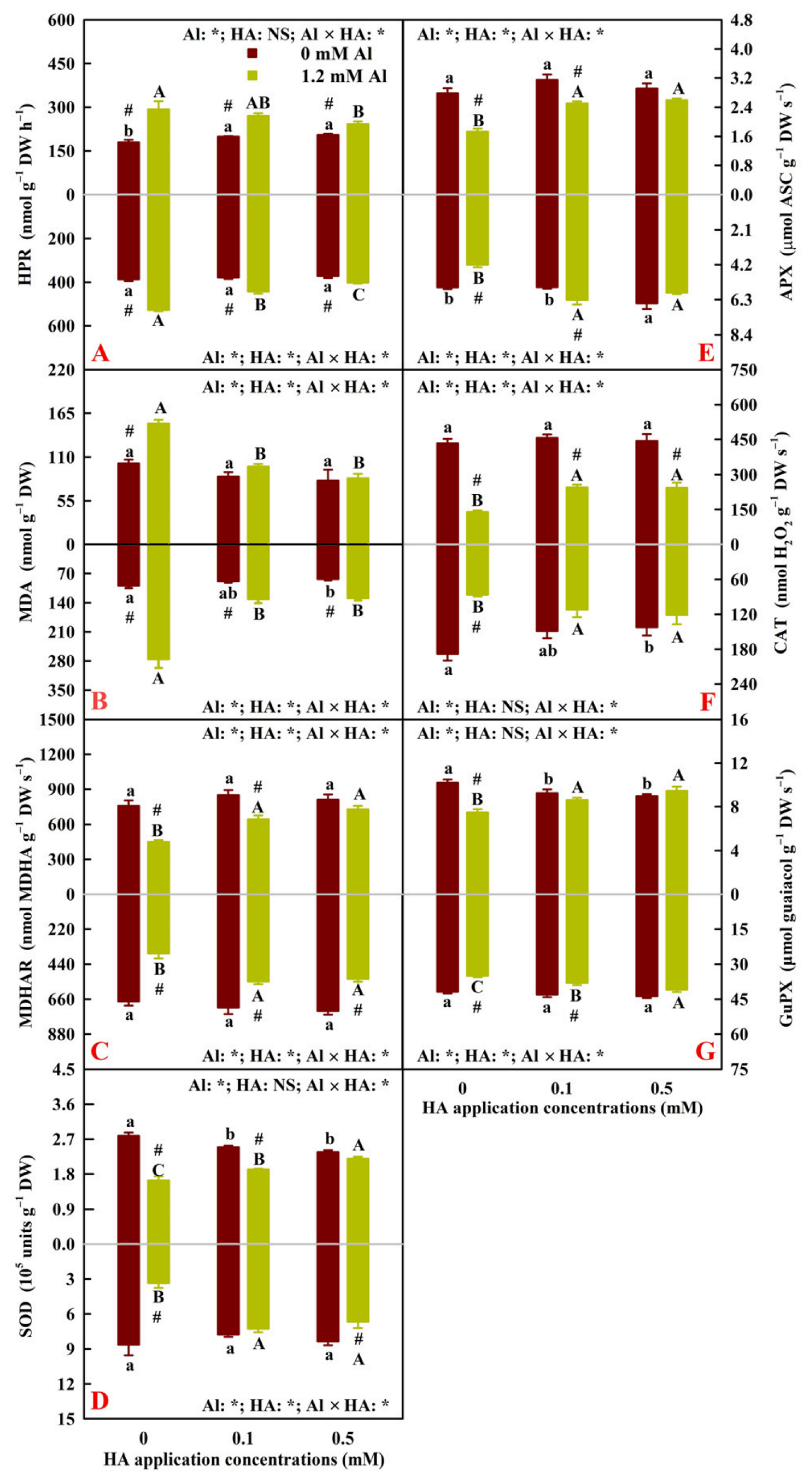
Impacts of Al-HA treatments on the mean (± SE, *n* = 4) HPR (**A**), concentrations of MDA (**B**), and activities of MDHAR (**C**), SOD (**D**), APX (**E**), CAT (**F**), and GuPX (**G**) in leaves (above column) and roots (below column). Bars with different lowercase and capital letters indicate significant differences at *p* ≤ 0.05 among three HA treatments within 0 and 1.2 mM Al, respectively. Bars with # indicate significant differences at *p* ≤ 0.05 between 0 and 1.2 mM Al treatments with the same HA treatment. NS, non-significant difference (*p* > 0.05); *, significant difference at *p* ≤ 0.05. APX, ascorbate (ASC) peroxidase; CAT, catalase; GuPX, guaiacol peroxidase; HA, humic acid; HPR, H_2_O_2_ production rate; MDA, malondialdehyde; MDHAR, monodehydroascorbate (MDHA) reductase; SOD, superoxide dismutase.

Supply of HA attenuated Al1.2-triggered rises in HPR and MDA concentrations and decreases in five antioxidant enzyme activities of leaves and roots. At Al1.2, the addition of HA decreased MDA concentrations, whereas it increased five antioxidant enzyme activities of leaves and roots. Root and leaf HPR decreased with the rise of HA supply. At Al0, the addition of HA did not change MDA concentration and MDHAR, APX and CAT activities in leaves, as well as HPR and MDHAR, SOD, and GuPX activities in roots; slightly decreased SOD and GuPX activities in leaves; and slightly elevated HPR in leaves. Root MDA concentration and CAT activity decreased with the increase in HA supply, and root APX activity was greater at HA0.5 than at HA0 and HA0.1 (Figure 11).

### 2.6. Principal Coordinate Analysis Plots, Pearson Correlation Coefficients (PCCs), and the Different Physiological Responses of Leaves and Roots to Al-HA Treatments

**Figure 12:**
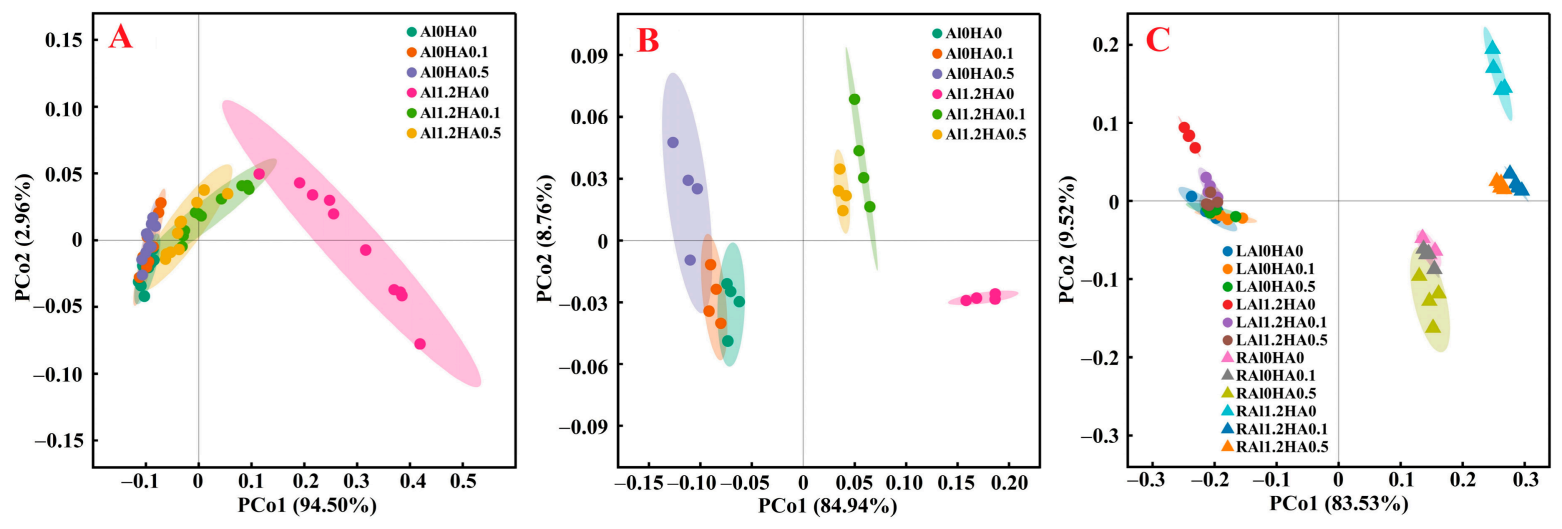
Principal coordinate analysis plots of 24 fluorescence and growth indexes (**A**) and another 160 indexes (**B**), as well as 44 common indexes present in both roots and leaves (**C**) from ‘Sour pummelo’ seedlings exposed to different Al-HA treatments. Al0HA0, 0 mM Al (Al0) + 0 mM HA (HA0); Al0HA0.1, Al0 + 0.1 mM HA (HA0.1); Al0HA0.5, Al0 + 0.5 mM HA (HA0.5); Al1.2HA0, 1.2 mM Al (Al1.2) + HA0; Al1.2HA0.1, Al1.2 + HA0.1; Al1.2HA0.5, Al1.2 + HA0.5; RAl0HA0, roots of Al0HA0-exposed seedlings; RAl0HA0.1, roots of Al0HA0.1-exposed seedlings; RAl0HA0.5, roots of Al0HA0.5-exposeed seedlings; RAl1.2HA0, roots of Al1.2HA0-exposed seedlings; RAl.2HA0.1, roots of Al1.2HA0.1-exposed seedlings; RAl.2HA0.5, roots of Al1.2HA0.5-exposed seedlings; LAl0HA0, leaves of Al0HA0-exposed seedlings; LAl0HA0.1, leaves of Al0HA0.1-exposed seedlings; LAl0HA0.5, leaves of Al0HA0.5-exposed seedlings; LAl1.2HA0, leaves of Al1.2HA0-exposed seedlings; LAl.2HA0.1, leaves of Al1.2HA0.1-exposed seedlings; LAl.2HA0.5, leaves of Al1.2HA0.5-exposted seedlings.

The PCoA was carried out using 24 fluorescence and growth indexes (Figure 12A) and another 160 indexes (Figure 12B) to understand their response patterns to Al-HA treatments. For these 24 (160) indexes, PCo1 and PCo2 explained 94.50% (84.94%) and 2.96% (8.77%) of the total variation, respectively. The PCo1 separated the effects of Al1.2 on these indexes and the influences of HA supply on these indexes at Al1.2. The PCoA suggested that the addition of HA inhibited Al1.2-triggered alterations of these indexes, as evidenced by the shorter distance between Al1.2HA0.5(0.1) and Al0HA0.5(0.1) than between Al1.2HA0 and Al0HA0. This agreed with our data that Al1.2 had no significant influence on 11 (three from Figure S2, one from Figure S3, four from Figure 6, one from Figure S5, and two from Figure 7), 17 (two from Figure 3, one from Figure S2, three from Figure S3, one from Figure 5, three from Figure 6, two from Figure S5, one from Figure 7, and four from Figure 11), and 25 (two from Figure 3, two from Figure S2, one from Figure S3, one from Figure 5, four from Figure 6, three from Figure 7, four from Figure 10, and eight from Figure 11) out of 184 indexes at HA0, HA0.1, and HA0.5, respectively; and that the addition of HA mitigated Al1.2-triggered changes in most indexes (Figure 1, Figure 2, Figure 3, Figure 4, Figure 5, Figure 6 and Figure 7, Figure 9, Figure 10 and Figure 11 and Figures S2–S5) and OJIP transients (Figure 8), as well as Al-toxic symptoms (Figure 1).

To reveal the relationship between different indicators, we used 184 indexes to calculate the PCCs (Table S1). This study also conducted a curve regression analysis between some parameters (Figure S6). There were many positive and negative relationships between various parameters. Further analysis showed that among the 45 common indexes present in both roots and leaves (44 common indexes for PCoA + root and leaf DW), 31 (27) leaf indexes displayed a positive (significant positive) relation to the corresponding root indexes, but only 14 (11) indexes exhibited a negative (significant negative) relation to the corresponding root indexes.

**Figure 13:**
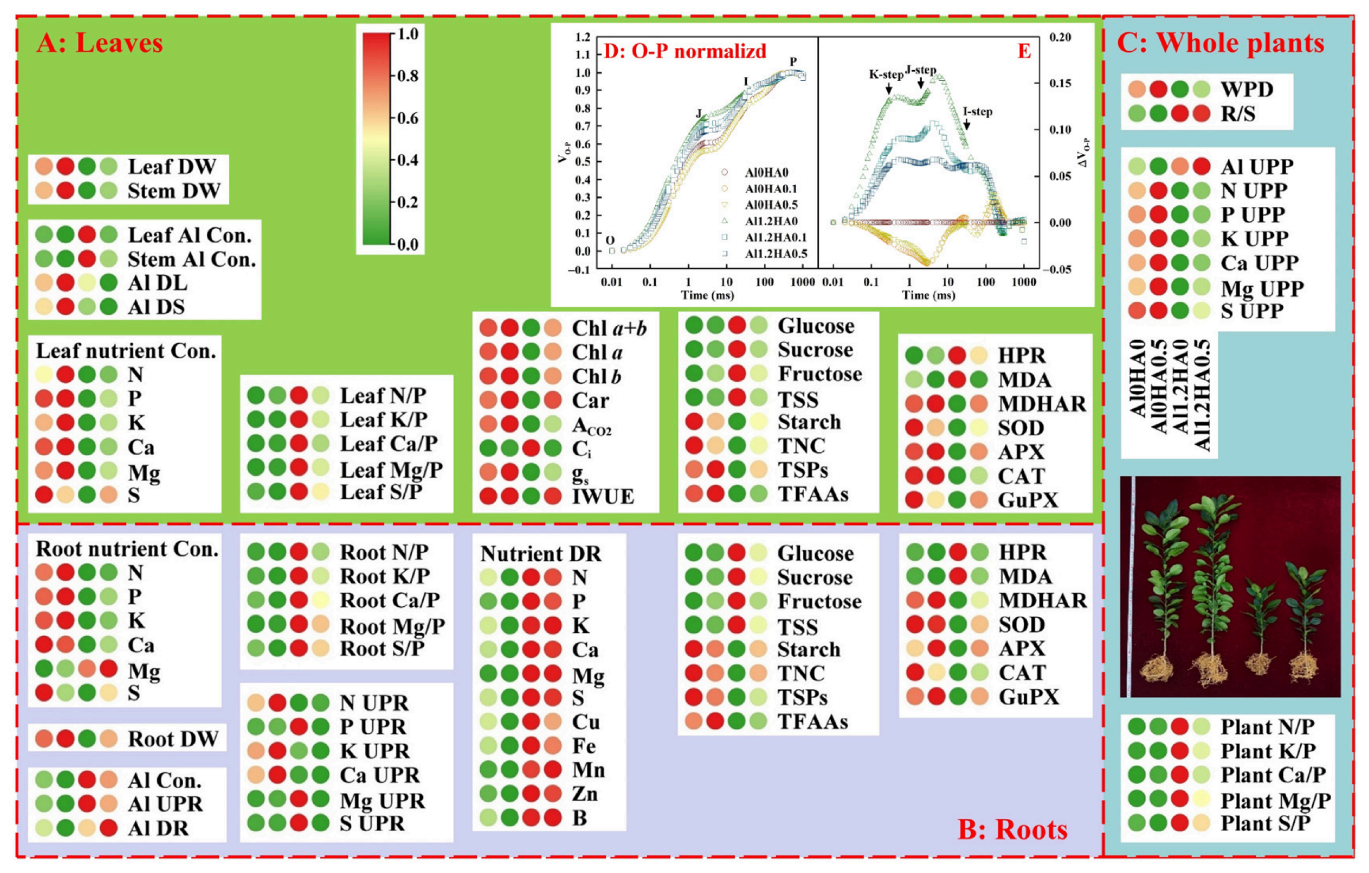
A diagram showing the impacts of HA-Al treatments on growth, nutrient uptake, gas exchange, and related physiological indexes in ‘Sour pummelo’ seedlings. Al0HA0, 0 mM Al (Al0) + 0 mM HA (HA0); Al0HA0.5, Al0 + 0.5 mM HA (HA0.5); Al1.2HA0, 1.2 mM Al (Al1.2) + HA0; Al1.2HA0.5, Al1.2 + HA0.5; Con., concentration; DL, distribution in leaves; DR, distribution in roots; DS, distribution in stems; HA, humic acid; WPD, whole plant DW. (**A**) all parameters were leaf parameters except for stem DW, stem Al Con., and Al DS; (**B**) root parameter; (**C**) seedling growth and whole plant parameters; (**D**,**E**) OJIP transients expressed as the kinetics of relative variable fluorescence: between F_o_ and F_m_: V_O-P_ = (F_t_ − F_o_)/(F_m_ − F_o_) (**D**) and the differences of the four samples to the reference sample treated with Al0COU0 (ΔV_O-P_, (**E**)).

The PCoA was made using the 44 common indexes present in both leaves and roots to reveal their differential responses to Al-HA treatments (Figure 12C). The PCoA showed that the six treatments for leaves (roots) were clustered on the left (right) side, revealing great differential responses of leaves and roots to Al-HA treatments. This was supported by the data that 11 (Al, K, S, Mg, N, P, Mn, Fe, Cu, B, and Zn distributions in roots) out of 44 indexes in roots displayed a significant negative relation to the corresponding indexes in leaves (Table S1). In addition, Al1.2 elevated (lessened) Ca distribution and Mg level in roots (leaves), as well as Fe level at HA0 in roots (leaves) (Figure 3, Figure 6 and Figure S2). Notably, the clustering degree of the six treatments for leaves was higher than that for roots (Figure 12C). This implied that Al-HA treatments affected the 44 common indexes more in roots than in leaves, which was in agreement with the data that Al1.2-treated seedlings retained most of the Al in their roots, and at Al1.2, the concentration of Al was far higher in roots than in leaves (Figure 2). This was also backed up by the data that Al1.2 had no significant impact on zero, two (from Figure 11), and five (two from Figure 10 and three from Figure 11) out of 44 root indexes at HA0, HA0.1, and HA0.5, respectively; and four (one from Figure S2, and three from Figure 6), six (one from Figure 3, one from Figure S2, one from Figure 5, one from Figure 6, and two from Figure 11), and 11 (one from Figure 3, one from Figure S2, one from Figure 5, one from Figure 6, two from Figure 10, and five from Figure 11) out of 44 leaf indexes at HA0, HA0.1, and HA0.5, respectively; and that among the 44 indexes, most parameters displayed a greater change in roots than in leaves (Figure 2, Figure 3, Figure 5, Figure 6, Figure 10, Figure 11 and Figure 13).

## 3. Discussion

### 3.1. Humic Acid-Mediated Mitigation of Al^3+^ Stress Involved Reduced Ability to Absorb Al and Less Root-to-Shoot Al Transport, as Well as Improved Nutrient Homeostasis at Al1.2

#### 3.1.1. Addition of HA Reduced Al UPR, Tissue Al Levels, and Root-to-Shoot Al Transport at Al1.2

Resilience to Al stress in plants is not only associated with less Al acquisition, but also with relatively less root-to-shoot Al transport [20,43]. The research indicated that in Al1.2-treated seedlings, the addition of HA reduced Al UPR, tissue Al concentrations, and root-to-shoot Al transport, whereas it increased Al UPP (Figure 2). Al^3+^ stress can directly inhibit cell division in the root apical meristem, leading to severe restriction of the root system [10]. Previous studies demonstrated that P- and B-mediated attenuation of Al^3+^ stress in pummelo seedlings involved lessened leaf and stem Al concentrations and augmented or unaltered root Al concentration [44,45]; and that S-mediated mitigation of Al^3+^ stress in pummelo seedlings involved decreased leaf Al levels and unaltered root Al levels [21]. Yan et al. [46] observed that the attenuation of Al^3+^ stress in trifoliate orange (*Poncirus trifoliata* (L.) Raf.) mediated by B involved reduced Al uptake brought about by increased root rhizosphere pH and promoted malate secretion. Yang et al. [20,47] showed that augmenting pH conferred ‘Xuegan’ seedlings Al resistance by reducing tissue Al concentrations and Al UPR due to increased exudation of root malate and citrate, rather than by reduced Al UPP and increased Al accumulation in roots. It was found that any one of five biomass parameters (whole plant, leaf, root, leaf, stem, and shoot DW) was significantly negatively related to Al UPR; whole plant DW was significantly negatively related to leaf, root, and stem Al concentrations; that stem (root) DW was significantly negatively related to stem (root) Al level; and leaf DW displayed a lessened trend with elevating leaf Al (*r* = −0.7827) (Table S1). Notably, leaf DW showed a significant curve decrease with increasing leaf Al (*y* = 5.6270 + 38.8987*e*^−0.0589*x*^, *r* = −0.9376, *p* = 0.0421). Taken together, the addition of HA might lower Al UPR, tissue Al concentrations, and root-to-shoot Al transport, thereby ameliorating Al1.2-triggered growth inhibition (Al^3+^ stress) in ‘Sour pummelo’ seedlings.

#### 3.1.2. Addition of HA Improved the Capability of Seedlings to Keep Macronutrient Homeostasis at Al1.2

In addition to having a direct influence on plant growth and development, HA can also enhance nutrient availability and uptake, thus promoting plant growth [48,49]. The functional (phenolic and carboxyl) groups in HA can form chelates with Al and Fe and solubilize insoluble phosphate (Pi) rock minerals (FePO_4_), thereby enhancing P availability [31]. Additionally, P is essential for ATP biosynthesis, and ATP provides energy for the transport of various nutrients against electrochemical gradients across the plasma membrane (PM) of roots [50]. Therefore, HA can also promote other nutrient uptake by improving P absorption and subsequent ATP biosynthesis. Rosolem et al. [51] indicated that HA augmented P transport in soils and P uptake by maize. Huang et al. [52] showed that the addition of HA promoted macronutrient UPR (UPP) in ‘Xuegan’ seedlings. The research showed that the addition of HA augmented macronutrient UPR (UPP) and their levels in roots, leaves, and stems with a few exceptions, and that addition of HA alleviated Al1.2-triggered decreases in macronutrient UPR (UPP) and their levels in roots, leaves, and stems with a few exceptions (Figure 3 and Figure 4). Linear regression analysis indicated that a significant positive relationship existed between any two of the 17 indexes (root, shoot, stem, leaf, and whole plant DW and Mg, Ca, S, N, P, and K UPR and UPP) (Table S1). Phosphorus starvation was the cause of the growth decrease in triticale (×*Triticosecale* Wittmack) under Al^3+^ stress [53]. Aluminum can substitute for Ca to disturb cell expansion [9]. The addition of S [21] and P [44] alleviated the growth reduction caused by Al^3+^ stress in pummelo seedlings. Previous studies on other plants indicated that Mg [14] and Ca [54] mitigated growth inhibition brought about by Al^3+^. It is known that amino acids (AAs), the fundamental building blocks for protein biosynthesis, have a critical role in plant growth, development, and stress resilience [55,56]. Nitrogen limitation decreased root and leaf TFAAs and TSPs [57]. It was observed that a significant positive relationship existed between any two of the four leaf (root) indexes (N level, root or leaf DW, TSP concentration, and TFAA concentration) (Table S1), implying that the addition of HA reduced Al1.2-triggered decreases in root and leaf N, TFAA, and TSP concentrations, thereby improving root and leaf growth at Al1.2. Plants under stress often face an energy deficit. The reductions in root and leaf TFAA and TSP concentrations induced by Al1.2 might contribute to energy homeostasis, because both AA and protein biosynthesis consume enormous energy. These findings suggested that the addition of HA alleviated Al1.2-triggered growth inhibition by augmenting the ability of roots to absorb macronutrients at Al1.2. This was backed up by the reports that both HA-mediated mitigation of growth inhibition in ‘Xuegan’ seedlings brought about by excessive Cu [52] and raised pH-mediated mitigation of growth inhibition in ‘Xuegan’ seedlings brought about by Al^3+^ [20] involved increased capacity of roots to absorb macronutrients.

It was observed that Al1.2-stimulated decreases in P UPP, UPR, and concentrations in tissues were greater than those of five other macronutrients (Figure 3) and subsequently augmented plant, UPR, and tissue leaf S/P, K/P, Mg/P, Ca/P and N/P (Figure 5 and Figure S4). Nutrient balance is essential for plant growth and stress resilience [58]. Evidence shows that nutritional imbalance can restrict plant growth [59,60]. Nitrate or P deprivation triggered a more pronounced molecular response than combined nitrate and P deprivation in chickpea (*Cicer arietinum* L.) roots and leaves [61]. Linear regression analysis revealed that any one of five biomass indexes was significantly negatively related to any one of 25 indexes (plant, UPR, and tissue N/P, S/P, Mg/P, Ca/P, and K/P) (Table S1). These results suggested that supplementation of HA augmented the ability of roots to acquire P and to keep the balance of macronutrients, thereby promoting seedling growth under Al^3+^ stress.

Like previous works [20,21,62], the current work demonstrated that Al1.2 led to a significant rise in R/S. However, the R/S of ‘Swingle’ citrumelo (*Citrus paradisi* Mcf. × *Poncirus trifoliata* Raf.) plants did not significantly alter in response to Al^3+^ [63]. Tóth et al. [64] revealed that Al^3+^ toxicity decreased the R/S of 23 out of 25 common bean (*Phaseolus vulgaris* L.) cultivars and did not significantly alter the R/S of another two cultivars (‘Arapaho’ and ‘AC Island’). Nutrient availability has a strong influence on root systems [65]. The increment of R/S induced by Al1.2 agreed with the view that in nutrient-poor conditions, underground competition dominates, and plants allocate a higher proportion of biomass to their roots to compensate for the shortage of underground nutrients or to seek nutrient-rich patches [66]. Using meta-analysis, Lopez et al. [65] revealed that nutrient (K, N, and P) limitation increased the R/S of arable crops, with influences reducing in the order: P deficiency > N deficiency > K deficiency. Yeh et al. [67] examined the impacts of nutrient deficiencies on R/S in *Spathiphyllum* spp. Distilled H_2_O treatment, N deprivation, and P deprivation augmented R/S, with impacts decreasing in the order: H_2_O > N deprivation > P deprivation; Mg and K deprivation did not significantly alter R/S, and Ca deprivation decreased R/S. It was observed that R/S in ‘Xuegan’ and pummelo seedlings was increased by B [68], N [60] and P starvation [69], and decreased by Mg starvation [70]. Linear regression analysis indicated that R/S significantly increased with decreasing any one of the macronutrient UPPs (UPR), as well as with augmenting the distribution of any one of the nutrients in roots (Table S1). These results indicated that Al^3+^ stress increased nutrient allocation to roots to support their growth, thereby increasing R/S, and that the increase might be an adaptation to Al^3+^ toxicity, because there were relatively more roots that provided nutrients to relatively fewer shoots.

Taken together, the addition of HA enhanced macronutrient absorption and balance, thus promoting seedling growth at Al1.2.

### 3.2. Humic Acid-Mediated Mitigation of Al^3+^ Toxicity Involved Enhanced Ability to Combat Oxidative Stress at Al1.2

Under normal conditions, the removal and production of ROS in plant cells are in a dynamic balance. Al^3+^ toxicity can disrupt the balance and lead to lipid peroxidation [22]. The research showed that the addition of HA reduced Al1.2-triggered rises in Al UPR, and root and leaf HPR and accumulation of MDA and Al, and reductions in root and leaf accumulation of TSPs (Figure 2 and Figure 10). A significant positive relationship existed between any two of the four indexes (Al UPR, Al concentration, MDA concentration, and HPR), and TSP concentration was significantly negatively related to any one of the above four indexes in leaves and roots, except for the correlations between leaf MDA concentration and leaf HPR (*r* = 0.6419), leaf Al concentration and leaf HPR (*r* = 0.7939), and Al UPR and leaf Al concentration (*r* = 0.8108) (Table S1). Further analysis indicated that leaf Al (MDA) concentration curvilinearly increased with increasing leaf HPR at *p* < 0.05, and that Al UPR curvilinearly increased with increasing leaf Al concentration at *p* < 0.05 (Figure S6). These findings implied that the addition of HA lessened Al1.2-stimulated rises in root and leaf Al concentrations and ROS generation, thereby contributing to the attenuation of oxidative damage and the increases in TSP concentrations.

The research revealed that the addition of HA lessened Al1.2-triggered declines in root and leaf SOD, APX, CAT, GuPX, and MDHAR activities and increases in HPR and MDA concentrations (Figure 11). It was found that in roots and leaves, the MDA level was significantly negatively related to MDHAR, APX, CAT, SOD, and GuPX activities (Table S1 and Figure S2). As the first line of defense for ROS removal, SOD catalyzes the conversion of superoxide anion to H_2_O_2_ and O_2_ [71]. In peroxisomes, H_2_O_2_ can be catalyzed into H_2_O by CAT, whereas in chloroplasts, H_2_O_2_ can be converted to H_2_O by APX using ASC as an electron donor. The yielded MDHA can be reduced to ASC by MDHAR [72]. Peroxidases (PODs) also play key roles in catalyzing the reduction of H_2_O_2_ using various electron donors [73]. Evidence shows that humic substances can endow plants with stress tolerance by activating antioxidant enzymes including APX, CAT, GuPX, and SOD [34,49]. Overexpression of a wheat (*Triticum aestivum* L.) *WMnSOD1* endowed canola (*Brassica napus* L.) plants with Al resilience [26]. Overexpression of an *Arabidopsis thaliana* (L.) Heynh. peroxidase gene (*AtPrx64*) in tobacco (*Nicotiana tabacum* L.) enhanced plant resistance to Al^3+^ toxicity by lowering root Al, H_2_O_2_, and MDA concentrations and augmenting root TSP concentration and PM H^+^-ATPase activity under Al^3+^ stress [27]. Overexpression of a maize Al tolerance 6 gene (*ZmAT6*) conferred maize plants Al resilience by enhancing the root SOD, GuPX, CAT, and APX activities and lowering the root MDA and Al concentrations [74]. Taken together, the addition of HA augmented leaf and root ability to resist oxidative stress brought about by Al^3+^ stress by lessening ROS formation and enhancing antioxidant enzyme activities.

Under stressed conditions, excessive accumulation of ROS can cause oxidative burst and impairment to biomolecules (lipids, DNA, and proteins), and negatively affect plant growth [75,76]. It was observed that leaf (root) DW was significantly negatively related to leaf (root) HPR, MDA concentration, and TSP concentration, and that leaf (root) TSP concentration was significantly negatively related to leaf (root) HPR and MDA concentration (Table S1 and Figure S6). These results implied that HA supplementation lessened the damage of Al1.2 to biomolecules by reducing oxidative stress, thereby improving plant growth at Al1.2.

### 3.3. Causes for HA-Mediated Amelioration of Leaf Chl Decline Caused by Al1.2

The research found that the addition of HA mitigated leaf Chl decline brought about by Al1.2 (Figure 7), and that leaf Chl *a + b* levels were significantly decreased with augmenting leaf Al levels (Table S2). A high concentration of Al^3+^ is harmful to Chl biosynthesis and can lead to Chl breakdown [77]. These findings suggested that the addition of HA mitigated the Al1.2-triggered decrease in Chl biosynthesis and increase in Chl degradation by lessening leaf Al levels, thereby preventing Chl decline at Al1.2. Linear regression analysis showed that the Chl *a + b* level was significantly positively related to leaf Mg level and Mg UPP (Table S1), suggesting that the addition of HA might mitigate the competition of Al with Mg, thereby preventing Al1.2-triggered Chl decline [9,77]. It was observed that the addition of P [44] and S [21] mitigated Al^3+^ toxicity-triggered Chl decline, and that both S and P deficiencies led to a decrement in leaf Chl concentration [69,78]. The research indicated that leaf Chl level was significantly positively related to leaf S concentration, leaf P concentration, S UPP, and P UPP (Table S1), implying that the addition of HA lessened Al1.2-triggered declines in leaf S and P UPP (concentrations), thereby augmenting leaf Chl biosynthesis and concentration at Al1.2.

Besides Mg, S, and P deficiencies, other nutrient (N, K, and Ca) deficiencies [72,79,80] can lead to leaf Chl decline. Additionally, Chl concentration can be reduced by nutrient imbalance [81]. It was observed that the addition of HA ameliorated Al1.2-induced reductions in N, K, and Ca UPP (Figure 4) and increases in leaf and plant K/P, S/P, Mg/P, Ca/P, and N/P (Figure 5), that leaf N, K, and Ca concentrations significantly elevated or exhibited an increased trend with elevating HA supplementation at Al1.2 (Figure 3), and that Chl *a + b* concentration was significantly positively related to leaf Ca concentration, N concentration, K concentration, Ca UPP, K UPP, and N UPP, but negatively to leaf and plant K/P, S/P, Mg/P, Ca/P, and N/P (Table S1 and Figure S6). These results suggested that addition of HA augmented N, Ca and K UPP and maintained plant (leaf) macronutrient balance at Al1.2, thereby augmenting leaf Chl biosynthesis and concentration.

Taken together, in addition to directly increasing Chl biosynthesis and decreasing Chl breakdown, the addition of HA augmented leaf Chl concentration by reducing leaf Al accumulation and maintaining plant (leaf) macronutrient homeostasis at Al1.2.

### 3.4. Causes for HA-Mediated Amelioration of Leaf A_CO2_ Decline Induced by Al1.2

The decline in leaf A_CO2_ caused by Al1.2 could not be explained by lessened g_s_ alone, since Al1.2 increased or had no impact on leaf C_i_ (Figure 7) and A_CO2_ displayed a decreased trend with augmenting C_i_ (Table S1). This agreed with the reports that Al-triggered reductions in leaf A_CO2_ in sorghum [15] and *Citrus* [8,20] were mainly brought about by non-stomatal factors. It was found that A_CO2_ was significantly positively related to Chl *a + b* level (Table S1). However, the mitigation of HA to leaf A_CO2_ decline at Al1.2 could not be attributed to increased Chl concentration, since the Al1.2-triggered decrease in Chl concentration was much smaller than that of A_CO2_ (Figure 7). Similar results have been observed on leaves of Al-stressed ‘Xuegan’ [20], pummelo [21], and ‘Cleopatra’ tangerine (*Citrus reshni* Hort. Ex Tanaka) [82] seedlings. This agreed with the data showing that the addition of HA mitigated Al1.2-triggered increases in DI_o_/RC and DI_o_/CS_o_, and that A_CO2_ was significantly negatively related to DI_o_/RC and DI_o_/CS_o_ (Table S1).

The present research, like that of previous researchers [20,47], indicated that Al1.2-triggered leaf A_CO2_ decline could not be attributed to feedback inhibition by carbohydrate accumulation, because Al1.2 caused a decrease in leaf TNC concentration (Figure 10). Notably, Al1.2 increased leaf and root soluble sugar concentrations, but decreased leaf and root starch and TNC concentrations. The addition of HA alleviated the changes in leaf and root levels of NCs caused by Al1.2. Al^3+^-induced increases in soluble sugar (glucose, fructose, and sucrose concentrations) and decreases in starch and TNC concentrations have also been obtained in ‘Xuegan’ leaves and roots [47]. Glucose can mitigate harmful impacts of abiotic stress by augmenting antioxidant and sugar accumulation [83]. Addition of glucose increased shoot and root growth of *Arabidopsis* seedlings exposed to Cd toxicity and reduced Cd toxicity-triggered leaf chlorosis by augmenting Cd fixation in roots and lowering root-to-shoot Cd transport (root Cd concentration) [84]. Supplementation of glucose to Cu-exposed cucumber (*Cucumis sativus* L.) seedlings enhanced seedling growth and A_CO2_ by reducing oxidative stress and improving photosynthetic enzyme activities and N, K, and P uptake [85]. Carbohydrates can serve as signal molecules, antioxidants, and energy. Soluble sugars can provide energy to combat oxidative stress brought about by metal toxicity, and starch will be rapidly hydrolyzed to soluble sugars when needed [86]. The study indicated that Al1.2 caused a rise in the accumulation of TSS and a decrement in the accumulation of starch in roots and leaves, especially at HA0 (Figure 10), implying that Al1.2 increased the hydrolysis of starch into soluble sugars to combat energy deficits and oxidative stress. The higher accumulation of TSS and hydrolysis of starch in roots and leaves induced by Al1.2 at HA0 was consistent with the elevated need for ROS removal and energy.

The research demonstrated that the addition of HA alleviated Al1.2-triggered photoinhibition of PSII [87], as revealed by less increased DI_o_/RC, decreased ET_o_/ABS and F_v_/F_m_, and changed OJIP transients caused by Al1.2 (Figure 8 and Figure 9). Further analysis showed that addition of HA reduced Al1.2-triggered alterations of all fluorescence indexes measured here, and A_CO2_ was significantly positively related to F_m_, F_v_, F_v_/F_o_, F_v_/F_m_, ET_o_/RC, RE_o_/RC, ET_o_/ABS, PI_abs,total_, ET_o_/TR_o_, RE_o_/ABS, and PI_abs_, but negatively related to the other seven fluorescence indexes (Table S1). Previous studies suggested that Al^3+^-induced A_CO2_ decline in ‘Sour pummelo’ leaves was mainly due to impaired PETC revealed by OJIP transients [88]; and S [21], P [44], B [45], and augmenting pH [20] lessened the reduction of A_CO2_ caused by Al^3+^ toxicity by preventing the impairment of Al^3+^ toxicity to PETC in *Citrus* leaves. Huang et al. [37,52] reported that supply of HA reduced Cu-triggered impairment of PETC and decline of A_CO2_ in ‘Xuegan’ leaves. Collectively, supply of HA ameliorated the damage of Al1.2 to PETC, thereby enhancing leaf photosynthetic electron transport capacity and A_CO2_ (Figure 7).

Evidence shows that both nutrient starvation and imbalance can cause PETC impairment and subsequent A_CO2_ decline [69,81,89,90]. The study indicated that the supply of HA mitigated the reductions in macronutrient UPP and their levels in leaves with a few exceptions (Figure 4), and the increases in plant and leaf K/P, S/P, Ca/P, Mg/P, and S/P (Figure 5) caused by Al1.2. Linear regression analysis showed that any one macronutrient UPP and their concentrations in leaves (plant and leaf K/P, S/P, Mg/P, Ca/P, and N/P) was significantly positively (negatively) related to any one of A_CO2_, F_v_/F_o_, F_m_, F_v_, RE_o_/RC, ET_o_/ABS, F_v_/F_m_, RE_o_/ABS, ET_o_/RC, ET_o_/TR_o_, PI_abs_, and PI_abs,total_, but negatively (positively) related to any one of the other seven fluorescence indexes, with a few exceptions between nutrient concentrations and A_CO2_ and between nutrient concentrations and fluorescence indexes (Table S1). Previous studies indicated that augmented pH-mediated mitigation of A_CO2_ decline and PETC impairment caused by Al^3+^ in ‘Xuegan’ leaves involved elevated capacity to absorb nutrients and to keep nutrient balance [20], and that supply of HA mitigated Cu toxicity-induced nutrient uptake reduction, nutrient imbalance, leaf PEPC impairment [52], and leaf A_CO2_ decline [37] in ‘Xuegan’ seedlings. These findings suggest that HA supplementation elevated the capacity of seedlings to keep macronutrient homeostasis at Al1.2, thus preventing Al1.2-triggered impairment of PETC and decline of A_CO2_ in leaves.

Considered together, the addition of HA enhanced leaf A_CO2_ at Al1.2 by mitigating Al1.2-stimulated leaf PETC impairment and enhancing the capacity of seedlings to keep macronutrient homeostasis at Al1.2.

## 4. Materials and Methods

### 4.1. Materials

The research was carried out at Fujian Agriculture and Forestry University (FAFU), Fuzhou, with an average annual sunshine hours of ~1600 h, temperature of ~20 °C, and relative humidity of ~76% [20]. ‘Sour pummelo’ seeds were harvested from our orchard located in Xiayang Village, Changlong Town, Lianjiang County, Fuzhou, China. Harvested seeds were sown in plastic trays containing sand. Six weeks after germination, uniformly sized seedlings were transported into 6 L pots (two seedlings to a pot) filled with sand [45,52]. These seedlings were planted in a greenhouse under natural conditions at FAFU, Fuzhou. Seven weeks after transplanting, the seedlings were irrigated with freshly prepared solutions containing 0 (HA0), 0.1 (HA0.1), or 0.5 (HA0.5) mM sodium humate (pH 4.2, adjusted by HCl) for the first day (500 mL pot^−1^), and then irrigated daily with the freshly prepared nutrition solutions (pH 4.2, adjusted by NaOH or HCl) from the second to fourth day until run off (~ 500 mL pot^−1^), followed by 31 cycles of HA treatments for 1 day and Al treatments for 3 days. The concentrations of HA and Al and the application times were chosen according to our previous studies [7,21,52]. The nutrient solutions included 1 mM Ca(NO_3_)_2_, 0.5 mM MgSO_4_, 0.1 mM KH_2_PO_4_, 1 mM KNO_3_, 20 μM Fe-EDTA, 10 μM H_3_BO_3_, 2 μM ZnSO_4_, 2 μM MnCl_2_, 0.5 μM CuSO_4_, 0.065 μM (NH_4_)_6_Mo_7_O_24_, and 1.2 mM (Al1.2 or Al^3+^ toxicity) or 0 mM AlCl_3_·6H_2_O [7,52]. There were 12 pots per treatment in a completely randomized design. After 32 cycles, ~5-mm-long root tips and recently fully expanded (about seven-week-old) leaves were used for all analyses except for Al and nutrients. After the measurements of leaf OJIP transients and gas exchange, 6-mm-diameter leaf discs and root tips (one plant pot^−1^) were harvested and frozen in liquid N_2_ and then stored at −80 °C until analyses of enzymes and metabolites were conducted. These seedlings from the same pots without taking leaf discs and root tips (one plant pot^−1^) were used to measure biomass, Al, nutrients, and HPR.

### 4.2. Chemicals

All chemicals utilized in this study were analytical reagents or the best commercially available grades. Humic acid, sodium salt (CAS: 91-64-5; analytical reagent; molecular formula: C_9_H_8_Na_2_O_4_; MW: 226.14; H102874-500g; and Lot#K2112176) were purchased from Shanghai Aladdin Biochemical Technology Co., Ltd., Shanghai, China.

### 4.3. Measurements of OJIP Transients and Gas Exchange

Leaf OJIP transients were measured with a Handy PEA (Hansatech Instruments Limited, Norfolk, UK) using 3 h dark-adapted plants at 25 °C [45]. Table S2 lists the extracted indexes, as well as the calculation of derived indexes from the extracted indexes [90]. Measurements of A_CO2_, T_r_, g_s_, and C_i_ were made with a CIRAS-2 portable photosynthesis system (PP Systems, Herts, UK) between 9:30 and 11:30 a.m. [45]. Leaf IWUE was defined as: IWUE (μmol CO_2_ mmol^−1^ H_2_O) = A_CO2_/T_r_ [37].

### 4.4. Determination of Biomass, Pigments, Al, and Nutrients

Biomass was measured after plants were dried to a constant weight at ~70 °C [17]. The concentrations of leaf pigments were measured spectrophotometrically after extraction with 80% (*v*/*v*) acetone [91]. The middle sections of stems, the fibrous roots, and the recent fully expanded mature leaves were taken for analyses of Al and nutrients [17]. Phosphorus, Mn, Fe, K, S, Zn, Cu, Mg, Ca, N, and B were assayed as described by Huang et al. [52]. Aluminum was determined with aluminon [92]. The calculation of nutrient distribution and uptake was made according to Huang et al. [52].

### 4.5. Assay of HPR, MDA Concentration, and Antioxidant Enzyme Activities

H_2_O_2_ production rate was assayed using guaiacol and horseradish POD (EC 1.11.1.7) [82]. The improved thiobarbituric acid-reactive-substances assay was used to estimate MDA concentration [93]. Antioxidant enzymes were extracted with 50 mM KH_2_PO_4_-KOH (pH 7.5) containing 0.1 mM EDTA-Na_2_, 0.3% Triton X-100, and 4% insoluble polyvinylpolypyrrolidone. After centrifugation at 13,000× *g* and 4 °C for 10 min, the supernatant was collected to assay the activities of antioxidant enzymes [72]. The activity of SOD (EC 1.15.1.1) was assayed as described by Giannopolitis and Ries [94]. The activities of APX (EC 1.11.1.11), MDHAR (EC 1.6.5.4), and CAT (EC 1.11.1.6) were measured as given by Chen and Cheng [72]. Guaiacol POD (EC 1.11.1.7) was determined as given by Huang et al. [37].

### 4.6. Assay of NC, TSP, and TFAA Concentrations

After extraction with 80% (*v*/*v*) ethanol, glucose, sucrose, and fructose concentrations in the supernatant were determined by enzymatic assay [95], and starch concentration in the residue was determined by anthrone [96]. After extraction with 10% (*v*/*v*) acetic acid, TFAA concentration in the supernatant was measured by the ninhydrin method [97]. After extraction with 50 mM Na_2_HPO_4_–KH_2_PO_4_ (pH 7.0), the concentration of TSPs was measured by Coomassie Blue [98].

### 4.7. Statistical Analysis

SPSS statistical software (version 17.0, IBM, NY, USA) was used to calculate PCCs. ChiPlot (https://www.chiplot.online/, accessed on 5 April 2026) was used for principal coordinate analysis (PCoA). Significant evaluation of all data was made with DPS 7.05 (Hangzhou Ruifeng Information Technology Co., Ltd., Hangzhou, China). Two-way ANOVA was used to test interactions between Al-HA treatments. An unpaired *t*-test was used for comparison between the means of two Al treatments within the same HA treatment. One-way ANOVA followed by LSD was applied for comparison among the means of three HA treatments within the same Al treatment.

## 5. Conclusions

The current results indicated that the addition of HA lowered roots’ ability to absorb Al and tissue (root, stem and leaf) Al concentrations and subsequently mitigated the impairment of Al^3+^ stress to root growth and function, thereby enhancing the ability of plants to maintain macronutrient (S, K, Mg, Ca, N, and P) homeostasis, and hence alleviating Al^3+^ stress-stimulated oxidative damage and inhibition of A_CO2_ and growth. This study comprehensively revealed the underlying mechanisms for HA-mediated amelioration of Al^3+^ stress in woody plants—gaining insight from growth, Al and nutrient absorption, oxidative stress, and photosynthesis and related physiological parameters—and provided a basis to develop new approaches for correcting Al^3+^ toxicity in woody plants (*Citrus*). Applying organic fertilizers may become a routine measure to protect woody plants (*Citrus*) from Al^3+^ toxicity in the future because the application of organic fertilizers is beneficial for the sustainability of the *Citrus* industry. Like natural humic substances, chemically synthesized humic substances can be used to protect woody plants from Al^3+^ toxicity in the future. Notably, sand cultivation studies may overestimate the phytotoxicity of Al^3+^ in actual soils due to soil microorganisms and the soil buffering effect. Therefore, further large-scale field studies are needed to precisely use organic fertilizers to correct Al^3+^ stress in *Citrus*.

## Data Availability

The original contributions presented in this study are included in the article/Supplementary Materials. Further inquiries can be directed to the corresponding authors.

## Supplementary Materials

The following supporting information can be downloaded at: https://www.mdpi.com/article/10.3390/plants15152370/s1, Figure S1: Leaf yellowing and necrosis occurred in leaves of Al1.2 + HA0-exposed seedlings; Figure S2: Impacts of Al-HA treatments on the mean (±SE, *n* = 4) concentrations of six micronutrients in leaves (A–E), stems (F–J), and roots (K–O); Figure S3: Impacts of Al-HA treatments on the mean (±SE, *n* = 4) micronutrient uptake per plant (UPP, (A–E)) and uptake per root dry weight (UPR, (F–J)); Figure S4: Impacts of Al-HA treatments on the mean (±SE, *n* = 4) ratios of N, K, Ca, Mg, and S concentrations to P concentration in stems (A–E), as well as ratios of N, K, Ca, Mg, and S uptake per root dry weight (UPR) to P UPR (F–J); Figure S5: Impacts of Al-HA treatments on the mean (±SE, *n* = 4) distributions of micronutrients in leaves (A–E), stems (F–J), and roots (K–O); Figure S6: Curve regression analysis between some parameters; Table S1: Pearson correlation coefficient matrix for the mean values of 184 physiological parameters in six Al-HA treatments; Table S2: Summary of parameters, formulae and their description using data extracted from chlorophyll (Chl) *a* (OJIP) transient.

## Abbreviations

The following abbreviations are used in this manuscript:

AAs	Amino acids
ABS/RC	Absorption flux per reaction center (RC) at *t* = 0
A_CO2_	CO_2_ assimilation
APX	Ascorbate peroxidase
ASC	Ascorbate
Car	Carotenoids
CAT	Catalase
Chl	Chlorophyll
C_i_	Intercellular CO_2_ concentration
C_i_/C_a_	C_i_ and intercellular/ambient CO_2_ concentration ratio
DI_o_/CS_o_	Dissipated energy flux per cross section at *t* = 0
DI_o_/RC	Dissipated energy flux per RC at *t* = 0
DW	Dry weight
ET_o_/ABS	Quantum yield for electron transport at *t* = 0
ET_o_/RC	Electron transport flux per RC at *t* = 0
ET_o_/TR_o_	Probability (at time 0) that a trapped exciton moves an electron into the electron transport chain beyond Q_A_^−^;
F_m_	Maximum fluorescence
F_o_	Minimum fluorescence
F_V_	Maximum variable fluorescence
F_v_/F_m_	Maximum quantum yield of primary photochemistry
F_v_/F_o_	Maximum primary yield of photochemistry of PSII
g_s_	Stomatal conductance
GuPX	Guaiacol peroxidase
HA	Humic acid
HPR	H_2_O_2_ production rate
MDA	Malondialdehyde
MDHA	Monodehydroascorbate
MDHAR	Monodehydroascorbate reductase
M_o_	Approximated initial slope (in ms^−1^) of the fluorescence transient *V* = *f(t)*
NCs	Nonstructural carbohydrates
OJIP transient	Chl *a* fluorescence transient
PCCs	Pearson correlation coefficients
PETC	Photosynthetic electron transport chain
PI_abs_	Performance index
PI_abs,total_	Total performance index
PM	Plasma membrane
POD	Peroxidase
PSI	Photosystem I
PSII	Photosystem II
RE_o_/ABS	Quantum yield for the reduction of end acceptors of PSI per photon absorbed at *t* = 0
RE_o_/RC	Reduction of end acceptors at PSI electron acceptor side per RC at *t* = 0
ROS	Reactive oxygen species
R/S	Root DW/shoot DW ratio
SOD	Superoxide dismutase
SOM	Soil organic matter
TFAAs	Total free amino acids
TNC	Total nonstructural carbohydrates
TSPs	Total soluble proteins
TSS	Total soluble sugars
UPP	Uptake per plant
UPR	Uptake per root dry weight
V_J_	Relative variable fluorescence at the J-step (2 ms)
V_I_	Relative variable fluorescence at the I-step (30 ms)

## References

[1] Huang C.F., Ma Y. (2025). Aluminum resistance in plants: A critical review focusing on STOP1. Plant Commun..

[2] Kochian L.V. (1995). Cellular mechanisms of aluminum toxicity and resistance in plants. Annu. Rev. Plant Biol..

[3] Wang F., Li X., Chen C., Zhao L., Wei Y. (2025). Mechanism of melatonin in alleviating aluminum toxicity in plants: A review. Biology.

[4] Guo J.H., Liu X.J., Zhang Y., Shen J.L., Han W.X., Zhang W.F., Christie P., Goulding K.W., Vitousek P.M., Zhang F.S. (2010). Significant acidification in major Chinese croplands. Science.

[5] Wu S., Liang S., Hu C., Tan Q., Zhang J., Dong Z. (2022). Ecological region division of soil based supplementary fertilization and decrement fertilization in China *Citrus* orchards. J. Huazhong Agri. Univ..

[6] Jiang D., Du S., Shi J., Xu H., Liu S., Han H., Xu Y., Wang H., Yan M., Huang X. (2025). Glutathione mitigates aluminum toxicity in root-apex transition zone of rice through reducing aluminum absorption and maintaining redox balance. Plant Physiol. Biochem..

[7] Yang H., Xie R.R., Xia T.T., Tong L.Y., Wu T., Ye X., Huang Z.R., Yang L.T., Chen L.S. (2026). Coumarin reduces aluminum-induced inhibition of growth and photosynthesis in *Citrus grandis* by reducing tissue Al concentration and maintaining nutrient and redox homeostasis. Plants.

[8] Pereira W.E., de Siqueira D.L., Martinez C.A., Puiatti M. (2000). Gas exchange and chlorophyll fluorescence in four *Citrus* rootstocks under aluminum stress. J. Plant Physiol..

[9] Yang M., Tan L., Xu Y., Zhao Y., Cheng F., Ye S., Jiang W. (2015). Effect of low pH and aluminum toxicity on the photosynthetic characteristics of different fast-growing *Eucalyptus* vegetatively propagated clones. PLoS ONE.

[10] Ohki K. (1986). Photosynthesis, chlorophyll, and transpiration responses in aluminum stressed wheat and sorghum. Crop Sci..

[11] Okla M.K., Javed S., Tahir M.F., Anas M., Saleem M.H., Ahmed T., Saleh I.A., Zomot N., Alwasel Y.A., Abdel-Maksoud M.A. (2025). Evaluating the potential of *Acinetobacter calcoaceticus* in alleviation of aluminium stress in *Triticum aestivum*. 3 Biotech..

[12] Simon L., Kieger M., Sung S.S., Smalley T.J. (1994). Aluminum toxicity in tomato. Part 2. Leaf gas exchange, chlorophyll content, and invertase activity. J. Plant Nutr..

[13] Nie G., Huang Y., Wang Y., He J., Zhang R., Yan L., Huang L., Zhang X. (2024). Physiological and comprehensive transcriptome analysis reveals distinct regulatory mechanisms for aluminum tolerance of *Trifolium repens*. Ecotoxicol. Environ. Saf..

[14] Lyu M., Liu J., Xu X., Liu C., Qin H., Zhang X., Tian G., Jiang H., Jiang Y., Zhu Z. (2023). Magnesium alleviates aluminum-induced growth inhibition by enhancing antioxidant enzyme activity and carbon-nitrogen metabolism in apple seedlings. Ecotoxicol. Environ. Saf..

[15] Peixoto P.H., Da Matta F.M., Cambraia J. (2002). Responses of the photosynthetic apparatus to aluminum stress in two sorghum cultivars. J. Plant Nutr..

[16] de Sousa A., AbdElgawad H., Fidalgo F., Teixeira J., Matos M., Tamagnini P., Fernandes R., Figueiredo F., Azenha M., Teles L.O. (2022). Subcellular compartmentalization of aluminum reduced its hazardous impact on rye photosynthesis. Environ. Pollut..

[17] Guo P., Qi Y.P., Cai Y.T., Yang T.Y., Yang L.T., Huang Z.R., Chen L.S. (2018). Aluminum effects on photosynthesis, reactive oxygen species and methylglyoxal detoxification in two *Citrus* species differing in aluminum tolerance. Tree Physiol..

[18] Moustaka J., Ouzounidou G., Sperdouli I., Moustakas M. (2018). Photosystem II is more sensitive than photosystem I to Al^3+^ induced phytotoxicity. Materials.

[19] Żurek G., Rybka K., Pogrzeba M., Krzyżak J., Prokopiuk K. (2014). Chlorophyll a fluorescence in evaluation of the effect of heavy metal soil contamination on perennial grasses. PLoS ONE.

[20] Yang T.Y., Cai L.Y., Qi Y.P., Yang L.T., Lai N.W., Chen L.S. (2019). Increasing nutrient solution pH alleviated aluminum-induced inhibition of growth and impairment of photosynthetic electron transport chain in *Citrus sinensis* seedlings. BioMed Res. Int..

[21] Guo P., Li Q., Qi Y.P., Yang L.T., Ye X., Chen H.H., Chen L.S. (2017). Sulfur-mediated-alleviation of aluminum-toxicity in *Citrus grandis* seedlings. Int. J. Mol. Sci..

[22] Giannakoula A., Moustakas M., Syros T., Yupsanis T. (2010). Aluminum stress induces up-regulation of an efficient antioxidant system in the Al-tolerant maize line but not in the Al-sensitive line. Environ. Exp. Bot..

[23] Yamamoto Y., Kobayashi Y., Devi S.R., Rikiishi S., Matsumoto H. (2002). Aluminum toxicity is associated with mitochondrial dysfunction and the production of reactive oxygen species in plant cells. Plant Physiol..

[24] Sivaguru M., Liu J., Kochian L.V. (2013). Targeted expression of *SbMATE* in the root distal transition zone is responsible for sorghum aluminum resistance. Plant J..

[25] Guo T.R., Yao P.C., Zhang Z.D., Wang J.J., Wang M. (2012). Involvement of antioxidative defense system in rice seedlings exposed to aluminum toxicity and phosphorus deficiency. Rice Sci..

[26] Basu U., Good A.G., Taylor G.J. (2001). Transgenic *Brassica napus* plants overexpressing aluminum-induced mitochondrial manganese superoxide dismutase cDNA are resistant to aluminum. Plant Cell Environ..

[27] Wu Y., Yang Z., How J., Xu H., Chen L., Li K. (2017). Overexpression of a peroxidase gene (*AtPrx64*) of *Arabidopsis thaliana* in tobacco improves plant’s tolerance to aluminum stress. Plant Mol. Biol..

[28] Sible C.N., Seebauer J.R., Below F.E. (2021). Plant biostimulants: A categorical review, their implications for row crop production, and relation to soil health indicators. Agronomy.

[29] Goswami L., Nath A., Sutradhar S., Bhattacharya S.S., Kalamdhad A., Vellingiri K., Kim K.H. (2017). Application of drum compost and vermicompost to improve soil health, growth, and yield parameters for tomato and cabbage plants. J. Environ. Manag..

[30] Liu Y., Gao L., Wang C., Fu Z., Chen R., Jiang W., Yin C., Mao Z., Wang Y. (2024). Biochar combined with humic acid improves the soil environment and regulate microbial communities in apple replant soil. Ecotoxicol. Environ. Saf..

[31] Canellas L.P., Olivares F.L., Aguiar N.O., Jones D.L., Nebbioso A., Mazzei P., Piccolo A. (2015). Humic and fulvic acids as biostimulants in horticulture. Sci. Horti..

[32] Tiwari J., Ramanathan A.L., Bauddh K., Korstad J. (2023). Humic substances: Structure, function and benefits for agroecosystems: A review. Pedosphere.

[33] Duan D., Tong J., Xu Q., Dai L., Ye J., Wu H., Xu C., Shi J. (2020). Regulation mechanisms of humic acid on Pb stress in tea plant (*Camellia sinensis* L.). Environ. Pollut..

[34] Antu U.B., Roy T.K., Kulsum T.I., Mitu P.R., Ismail Z., Arifin M., Datta M., Hossain S.A., Islam M.S., Mahiddin N.A. (2025). Role of humic acid for climate change adaptation measures to boost up sustainable agriculture and soil health: A potential review. Int. J. Biol. Macromol..

[35] Sayed A.A.S., Seoudi Z., Osman A.S., Semida W.M., Rady M.M., Elkelish A., Mahmoud A.E.M. (2024). Soil-plant integrative supplementation with humic acid and antioxidants improves growth, fruit quality, and antioxidant capacity of Cd-stressed *Solanum melongena*. J. Soil Sci. Plant Nutr..

[36] Odongkara P., Adhikari A., Kang S.-M., Benjamin A.Y., Lee I.-J. (2025). Mechanistic aspect of humic acid and *Pseudomonas nitroreducens* strain UF50 to alleviate nickel toxicity in soybean. Ind. Crop Prod..

[37] Huang W.T., Chen X.F., Huang W.L., Shen Q., Lu F., Lai N.W., Guo J., Yang L.T., Ye X., Chen L.S. (2025). Humic acid enhances antioxidant and glyoxalase systems to combat copper toxicity in *Citrus*. Agronomy.

[38] Jaros-Tsoj K., Sitko K., Rudnicka M., Sugier P., Jaroszuk-Ściseł J., Rostański A., Rineau F., Papazoglou E.G., Alexopoulou E., Vangronsveld J. (2025). Beneficial effects of commercially available preparations of humic substances and mycorrhiza on growth and photosynthesis of sorghum and hemp cultivated on a metal(loid)-polluted field. Plant Soil.

[39] Ozfidan-Konakci C., Yildiztugay E., Bahtiyar M., Kucukoduk M. (2018). The humic acid-induced changes in the water status, chlorophyll fluorescence and antioxidant defense systems of wheat leaves with cadmium stress. Ecotoxicol. Environ. Saf..

[40] Diatloff E., Harper S.M., Asher C.J., Smith F.W. (1998). Effects of humic and fulvic acids on the rhizotoxicity of lanthanum and aluminium to corn. Aust. J. Soil Res..

[41] Harper S., Menzies N. (2023). Phytotoxic effects of Al on root growth are confounded in the presence of fulvic and humic acids. Soil Syst..

[42] Tan K.H., Binger A. (1986). Effect of humic acid on aluminum toxicity in corn plants. Soil Sci..

[43] Vose P.B., Randall P.J. (1962). Resistance to aluminium and manganese toxicities in plants related to variety and cation-exchange capacity. Nature.

[44] Jiang H.X., Tang N., Zheng J.G., Li Y., Chen L.S. (2009). Phosphorus alleviates aluminum-induced inhibition of growth and photosynthesis in *Citrus grandis* seedlings. Physiol. Plant..

[45] Jiang H.X., Tang N., Zheng J.G., Chen L.S. (2009). Antagonistic actions of boron against inhibitory effects of aluminum toxicity on growth, CO_2_ assimilation, ribulose-1,5-bisphosphate carboxylase/oxygenase, and photosynthetic electron transport probed by the JIP-test, of *Citrus grandis* seedlings. BMC Plant Biol..

[46] Yan L., Li S., Cheng J., Liu Y., Liu J., Jiang C. (2022). Boron contributes to excessive aluminum tolerance in trifoliate orange (*Poncirus trifoliata* (L.) Raf.) by inhibiting cell wall deposition and promoting vacuole compartmentation. J. Hazard. Mater..

[47] Yang T.Y., Qi Y.P., Huang H.Y., Wu F.L., Huang W.T., Deng C.L., Yang L.T., Chen L.S. (2020). Interactive effects of pH and aluminum on the secretion of organic acid anions by roots and related metabolic factors in *Citrus sinensis* roots and leaves. Environ. Pollut..

[48] Deng A., Wu X., Su C., Zhao M., Wu B., Luo J. (2021). Enhancement of soil microstructural stability and alleviation of aluminium toxicity in acidic latosols via alkaline humic acid fertiliser amendment. Chem. Geol..

[49] Jmaili K., Asbai Z., Waddi K., Bahlaouan B., Silkina A., Boutaleb N. (2025). Non-microbial biostimulants for plant growth and abiotic stress mitigation: A review of recent scientific innovations. Int. J. Environ. Stud..

[50] Muratore C., Espen L., Prinsi B. (2021). Nitrogen uptake in plants: The plasma membrane root transport systems from a physiological and proteomic perspective. Plants.

[51] Rosolem C.A., Nascimento C.A., Bertolino K.M., Picoli L.B. (2024). Humic acid enhances phosphorus transport in soil and uptake by maize. J. Plant Nutr. Soil Sci..

[52] Huang W.T., Shen Q., Yang H., Chen X.F., Huang W.L., Wu H.X., Lai N.W., Yang L.T., Huang Z.R., Chen L.S. (2024). Effects of humic acid-copper interactions on growth, nutrient absorption, and photosynthetic performance of *Citrus sinensis* seedlings in sand culture. J. Plant Growth Regul..

[53] Quartin V.L., Azinheira H.G., Nunes M.A. (2001). Phosphorus deficiency is responsible for biomass reduction of triticale in nutrient with aluminum. J. Plant Nutr..

[54] Lan T., You J., Kong L., Yu M., Liu M., Yang Z. (2016). The interaction of salicylic acid and Ca^2+^ alleviates aluminum toxicity in soybean (*Glycine max* L.). Plant Physiol. Biochem..

[55] Ahmad M.H., Zafar Z., Bibi H., Latif N., Afzal B., Ali N., Nisa Z.U., Usman S., Shah A.A., Shafique S. (2025). Effect of exogenously applied amino acids on photosynthetic pigments, metabolites and enzymatic antioxidants in *Zea mays* L. subjected to salt stress. Sci. Rep..

[56] Shahrajabian M.H., Cheng Q., Sun W. (2022). The effects of amino acids, phenols and protein hydrolysates as biostimulants on sustainable crop production and alleviated stress. Recent Pat. Biotechnol..

[57] Huang W.T., Zheng Z.C., Hua D., Chen X.F., Zhang J., Chen H.H., Ye X., Guo J.X., Yang L.T., Chen L.S. (2022). Adaptive responses of carbon and nitrogen metabolisms to nitrogen-deficiency in *Citrus sinensis* seedlings. BMC Plant Biol..

[58] Hafeez A., Rasheed R., Ashraf M.A., Rizwan M., Ali S. (2022). Effects of exogenous taurine on growth, photosynthesis, oxidative stress, antioxidant enzymes and nutrient accumulation by *Trifolium alexandrinum* plants under manganese stress. Chemosphere.

[59] Feil S.B., Pii Y., Valentinuzzi F., Tiziani R., Mimmo T., Cesco S. (2020). Copper toxicity affects phosphorus uptake mechanisms at molecular and physiological levels in *Cucumis* sativus plants. Plant Physiol. Biochem..

[60] Huang W.T., Xie Y.Z., Chen X.F., Zhang J., Chen H.H., Ye X., Guo J., Yang L.T., Chen L.S. (2021). Growth, mineral nutrients, photosynthesis and related physiological parameters of *Citrus* in response to nitrogen deficiency. Agronomy.

[61] Nasr Esfahani M., Inoue K., Nguyen K.H., Chu H.D., Watanabe Y., Kanatani A., Burritt D.J., Mochida K., Tran L.P. (2021). Phosphate or nitrate imbalance induces stronger molecular responses than combined nutrient deprivation in roots and leaves of chickpea plants. Plant Cell Environ..

[62] Graham C.J. (2001). The influence of nitrogen source and aluminum on growth and elemental composition of ‘Nemaguard’ peach seedlings. J. Plant Nutr..

[63] dos Santos C.H., Filho H.G., Rodrigues J.D., de Pinho S.Z. (2000). Influence of different levels of aluminum on the development of *Citrus* rootstock ‘Swingle’ citrumelo (*Citrus paradisi* Mcf. × *Poncirus trifoliata* Raf.) in nutrient solution. Braz. Arch. Biol. Techn..

[64] Tóth B., Moloi M.J., Szőke L., Danter M., Grusak M.A. (2021). Cultivar differences in the biochemical and physiological responses of common beans to aluminum stress. Plants.

[65] Lopez G., Ahmadi S.H., Amelung W., Athmann M., Ewert F., Gaiser T., Gocke M.I., Kautz T., Postma J., Rachmilevitch S. (2023). Nutrient deficiency effects on root architecture and root-to-shoot ratio in arable crops. Front. Plant Sci..

[66] Mašková T., Herben T. (2018). Root:shoot ratio in developing seedlings: How seedlings change their allocation in response to seed mass and ambient nutrient supply. Ecol. Evol..

[67] Yeh D.M., Lin L., Wright C.J. (2000). Effects of mineral nutrient deficiencies on leaf development, visual symptoms and shoot-root ratio of *Spathiphyllum*. Sci. Horti..

[68] Han S., Chen L.S., Jiang H.X., Smith B.R., Yang L.T., Xie C.Y. (2008). Boron deficiency decreases growth and photosynthesis, and increases starch and hexoses in leaves of citrus seedlings. J. Plant Physiol..

[69] Meng X., Chen W.W., Wang Y.Y., Huang Z.R., Ye X., Chen L.S., Yang L.T. (2021). Effects of phosphorus deficiency on the absorption of mineral nutrients, photosynthetic system performance and antioxidant metabolism in *Citrus grandis*. PLoS ONE.

[70] Yang G.H., Yang L.T., Jiang H.X., Wang P., Chen L.S. (2012). Physiological impacts of magnesium-deficiency in citrus seedlings: Photosynthesis, antioxidant system and carbohydrates. Trees Struct. Funct..

[71] Kim J.S., Kim H., Yim B., Rhee J.S., Won E.J., Lee Y.M. (2018). Identification and molecular characterization of two Cu/Zn-SODs and Mn-SOD in the marine ciliate *Euplotes crassus*: Modulation of enzyme activity and transcripts in response to copper and cadmium. Aquat. Toxicol..

[72] Chen L.S., Cheng L. (2003). Both xanthophyll cycle-dependent thermal dissipation and the antioxidant system are up-regulated in grape (*Vitis labrusca* L cv Concord) leaves in response to N limitation. J. Exp. Bot..

[73] Teng K., Xiao G.Z., Guo W.E., Yuan J.B., Li J., Chao Y.H., Han L.B. (2016). Expression of an alfalfa (*Medicago sativa* L.) peroxidase gene in transgenic Arabidopsis thaliana enhances resistance to NaCl and H_2_O_2_. Genet. Mol. Res..

[74] Du H., Huang Y., Qu M., Li Y., Hu X., Yang W., Li H., He W., Ding J., Liu C. (2020). A maize *ZmAT6* gene confers aluminum tolerance via reactive oxygen species scavenging. Front. Plant Sci..

[75] Meriga B., Reddy B.K., Rao K.R., Reddy L.A., Kishor P.B. (2004). Aluminium-induced production of oxygen radicals, lipid peroxidation and DNA damage in seedlings of rice (*Oryza sativa*). J. Plant Physiol..

[76] Zhao L., Bai T., Wei H., Gardea-Torresdey J.L., Keller A., White J.C. (2022). Nanobiotechnology-based strategies for enhanced crop stress resilience. Nat. Food.

[77] Cunha Neto A.R.D., Ambrósio A.D.S., Wolowski M., Westin T.B., Govêa K.P., Carvalho M., Barbosa S. (2020). Negative effects on photosynthesis and chloroplast pigments exposed to lead and aluminum: A meta-analysis. Cerne.

[78] Broadley M., Brown P., Cakmak I., Rengel Z., Zhao F., Marschner P. (2012). Function of nutrients: Micronutrients. Marschner’s Mineral Nutrition of Higher Plants.

[79] Duan S., Zhang C., Song S., Ma C., Zhang C., Xu W., Bondada B., Wang L., Wang S. (2022). Understanding calcium functionality by examining growth characteristics and structural aspects in calcium-deficient grapevine. Sci. Rep..

[80] Fu Y., Mason A.S., Song M., Ni X., Liu L., Shi J., Wang T., Xiao M., Zhang Y., Fu D. (2023). Multi-omics strategies uncover the molecular mechanisms of nitrogen, phosphorus and potassium deficiency responses in *Brassica napus*. Cell. Mol. Biol. Lett..

[81] Qian S.Y., Wang Q., Liu N., Quan L., Lei Z.F., Song X.Z., Ai J.G. (2018). Effects of nitrogen deposition and phosphorus addition on photosynthesis and chlorophyll fluorescence characteristics of Chinese fir. Ecol. Sci..

[82] Chen L.S., Qi Y.P., Liu X.H. (2005). Effects of aluminum on light energy utilization and photoprotective systems in *Citrus* leaves. Ann. Bot..

[83] Siddiqui H., Sami F., Hayat S. (2020). Glucose: Sweet or bitter effects in plants-a review on current and future perspective. Carbohydr. Res..

[84] Shi Y.Z., Zhu X.F., Wan J.X., Li G.X., Zheng S.J. (2015). Glucose alleviates cadmium toxicity by increasing cadmium fixation in root cell wall and sequestration into vacuole in *Arabidopsis*. J. Integr. Plant Biol..

[85] Yusuf M., Saeed Almehrzi A.S., Nasir Alnajjar A.J., Alam P., Elsayed N., Khalil R., Hayat S. (2021). Glucose modulates copper induced changes in photosynthesis, ion uptake, antioxidants and proline in *Cucumis sativus* plants. Carbohydr. Res..

[86] Wan H., Du J., He J., Lyu D., Li H. (2019). Copper accumulation, subcellular partitioning and physiological and molecular responses in relation to different copper tolerance in apple rootstocks. Tree Physiol..

[87] Force L., Critchley C., van Rensen J.J.S. (2003). New fluorescence parameters for monitoring photosynthesis in plants. Photosynth. Res..

[88] Jiang H.X., Chen L.S., Zheng J.G., Han S., Tang N., Smith B.R. (2008). Aluminum-induced effects on photosystem II photochemistry in *Citrus* leaves assessed by the chlorophyll a fluorescence transient. Tree Physiol..

[89] Hao J., Han H., Liu Y., Li J., Yang J., Ren B., Bai L. (2003). Phosphorus addition alleviates the inhibition of nitrogen deposition on photosynthesis of *Potentilla tanacetifolia*. Front. Environ. Sci..

[90] Kalaji H.M., Bąba W., Gediga K., Goltsev V., Samborska I.A., Cetner M.D., Dimitrovam S., Piszcz U., Bielecki K., Karmowska K. (2018). Chlorophyll fluorescence as a tool for nutrient status identification in rapeseed plants. Photosynth. Res..

[91] Lichtenthaler H.K. (1987). Chlorophylls and carotenoids: Pigments of photosynthetic biomembranes. Method. Enzymol..

[92] Hsu P.H. (1963). Effect of initial pH, phosphate, and silicate on the determination of aluminum with aluminon. Soil Sci..

[93] Hodges D.M., DeLong J.M., Forney C.F., Prange R.K. (1999). Improving the thiobarbituric acid-reactive-substances assay for estimating lipid peroxidation in plant tissues containing anthocyanin and other interfering compounds. Planta.

[94] Giannopolitis C.N., Ries S.K. (1977). Superoxide dismutases I. Occurrence in higher plants. Plant Physiol..

[95] Jones M.G.K., Outlaw W.J., Lowry O.H. (1977). Enzymic assay of 10^−7^ to 10^−14^ moles of sucrose in plant tissues. Plant Physiol..

[96] Yemm E.W., Willis A.J. (1954). The estimation of carbohydrates in plant extracts by anthrone. Biochem. J..

[97] Li C.P., Qi Y.P., Zhang J., Yang L.T., Wang D.H., Ye X., Lai N.W., Tan L.L., Lin D., Chen L.S. (2017). Magnesium-deficiency-induced alterations of gas exchange, major metabolites and key enzymes differ among roots, and lower and upper leaves of *Citrus sinensis* seedlings. Tree Physiol..

[98] Bradford M.M. (1976). A rapid and sensitive method for the quantitation of microgram quantities of protein utilizing the principle of protein-dye binding. Anal. Biochem..

