# Supplementary material for: Humic Acid Alleviates Aluminum Toxicity in Citrus grandis (L.) Osbeck: Insight from Growth, Gas Exchange, and Related Physiological Parameters
Source: Plants (Basel). 2026 Jul 31;15(15):2370. doi: 10.3390/plants15152370 (PMC13467780; doi:10.3390/plants15152370)
Supplement: Supplementary file 1 [file plants-15-02370-s001.zip › Supplementary Figures.pdf]

Supplementary Figures S1-S6

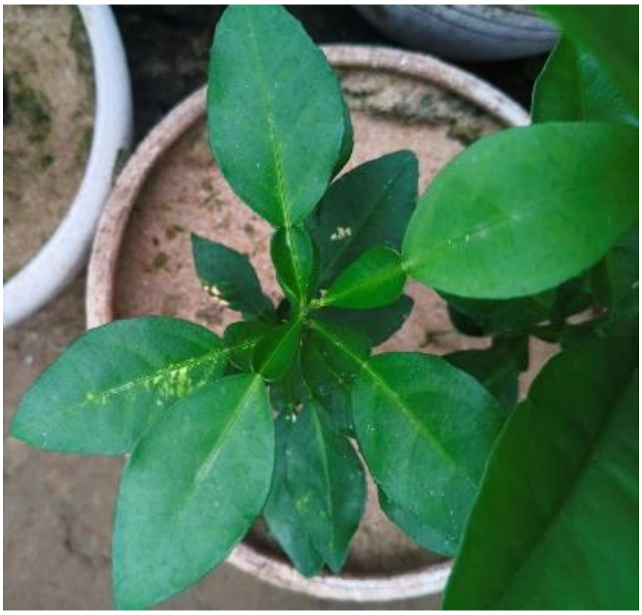

Figure S1. Leaf yellowing and necrosis occurred in leaves of Al1.2 + HA0-exposed seedlings

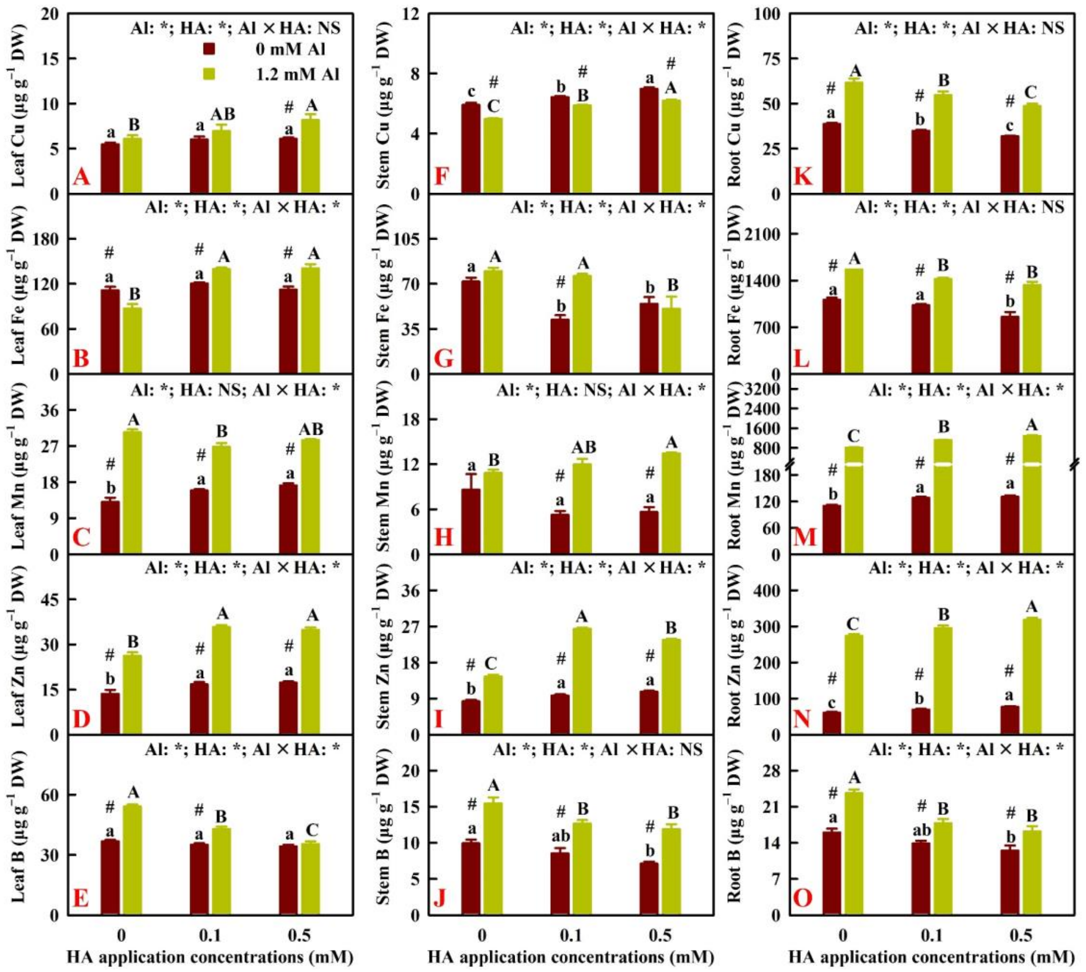

Figure S2. Impacts of Al-HA treatments on the mean (± SE, n = 4) concentrations of six micronutrients in leaves (**A-E**), stems (**F-J**), and roots (**K-O**). Bars with different lowercase and capital letters indicate significant different at $p \leq 0.05$ among three HA treatments within 0 and 1.2 mM Al, respectively. Bars with # indicate significant different at $p \leq 0.05$ between 0 and 1.2 mM Al treatments within the same HA treatment. NS, non-significant difference ($p > 0.05$); *, significant difference at $p \leq 0.05$. HA, humic acid.

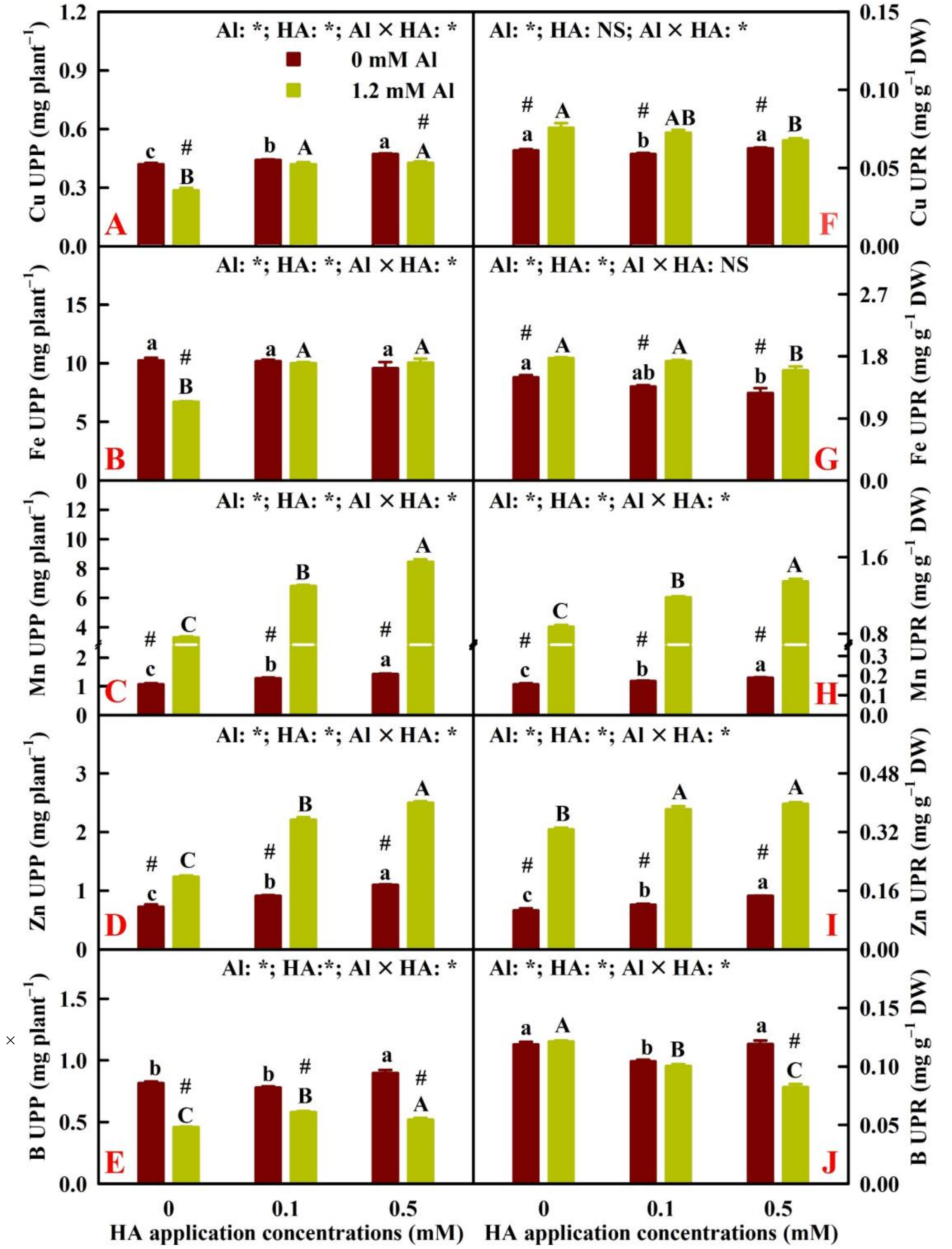

Figure S3. Impacts of Al-HA treatments on the mean (± SE, $n = 4$) micronutrient uptake per plant (UPP, (**A-E**)) and uptake per root dry weight (UPR, (**F-J**)). Bars with different lowercase and capital letters indicate significant different at $p \leq 0.05$ among three HA treatments within 0 and 1.2 mM Al, respectively. Bars with # indicate significant different at $p \leq 0.05$ between 0 and 1.2 mM Al treatments within the same HA treatment. NS, non-significant difference ($p > 0.05$); *, significant difference at $p \leq 0.05$. HA, humic acid.

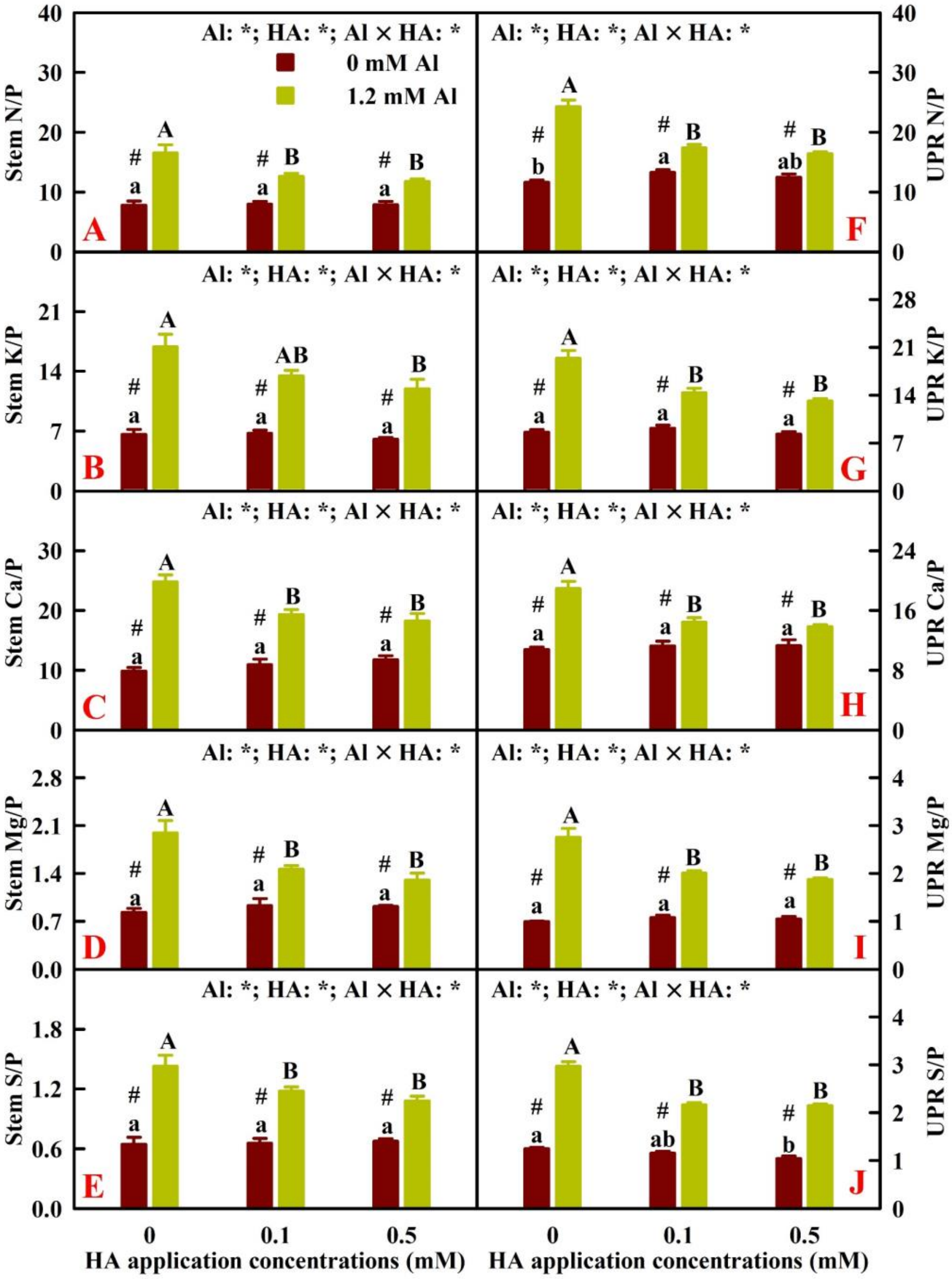

Figure S4. Impacts of Al-HA treatments on the mean (± SE, $n = 4$) ratios of N, K, Ca, Mg, and S concentrations to P concentration in stems (**A-E**), as well as ratios of N, K, Ca, Mg, and S uptake per root dry weight (UPR) to P UPR (**F-J**). Bars with different lowercase and capital letters indicate significant different at $p \leq 0.05$ among three HA treatments within 0 and 1.2 mM Al, respectively. Bars with # indicate significant different at $p \leq 0.05$ between 0 and 1.2 mM Al within the same HA treatment. *, significant difference at $p \leq 0.05$. HA, humic acid.

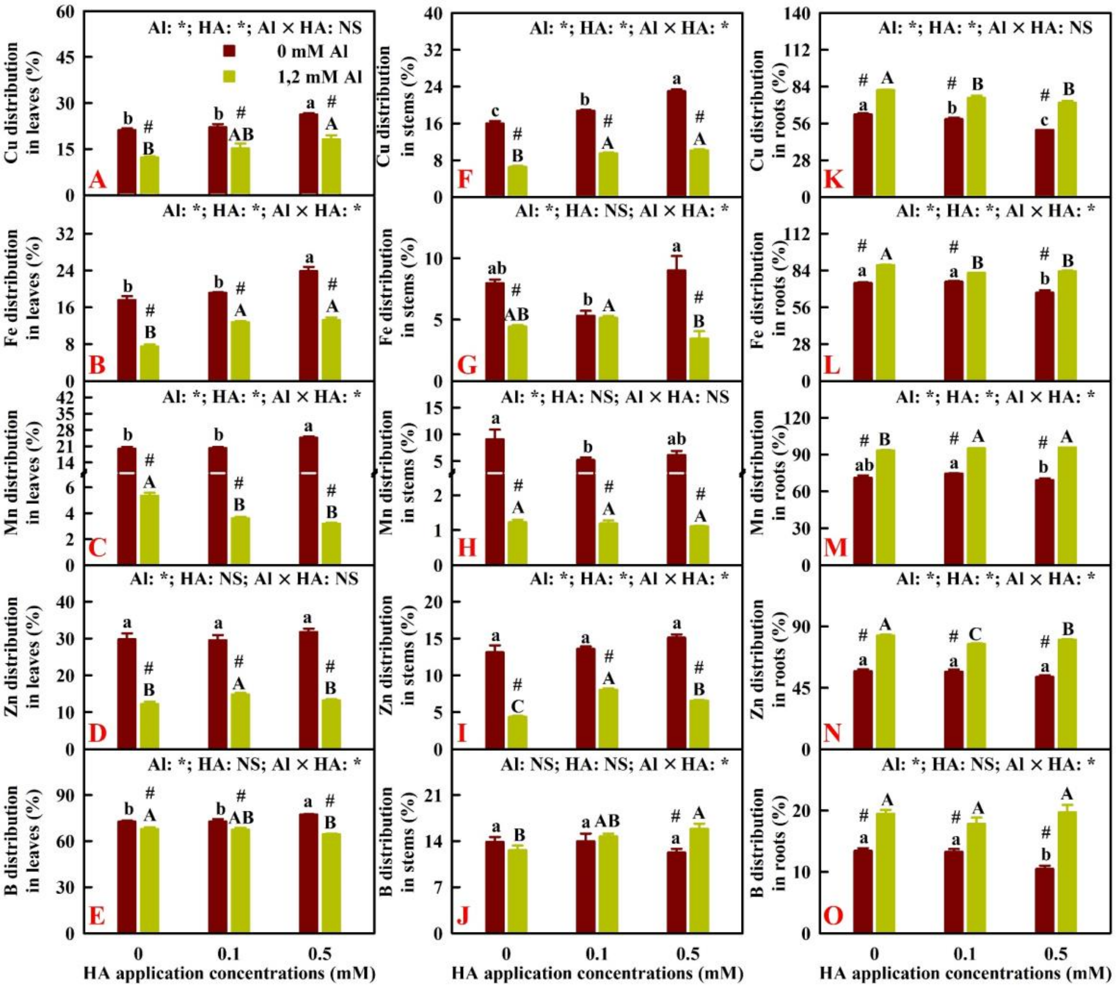

Figure S5. Impacts of Al-HA treatments on the mean (± SE, $n = 4$) distributions of micronutrients in leaves (**A-E**), stems (**F-J**), and roots (**K-O**). Bars with different lowercase and capital letters indicate significant different at $p \leq 0.05$ among three HA treatments within 0 and 1.2 mM Al, respectively. Bars with # indicate significant different at $p \leq 0.05$ between 0 and 1.2 mM Al treatments within the same HA treatment. NS, non-significant difference ($p > 0.05$); *, significant difference at $p \leq 0.05$. HA, humic acid.

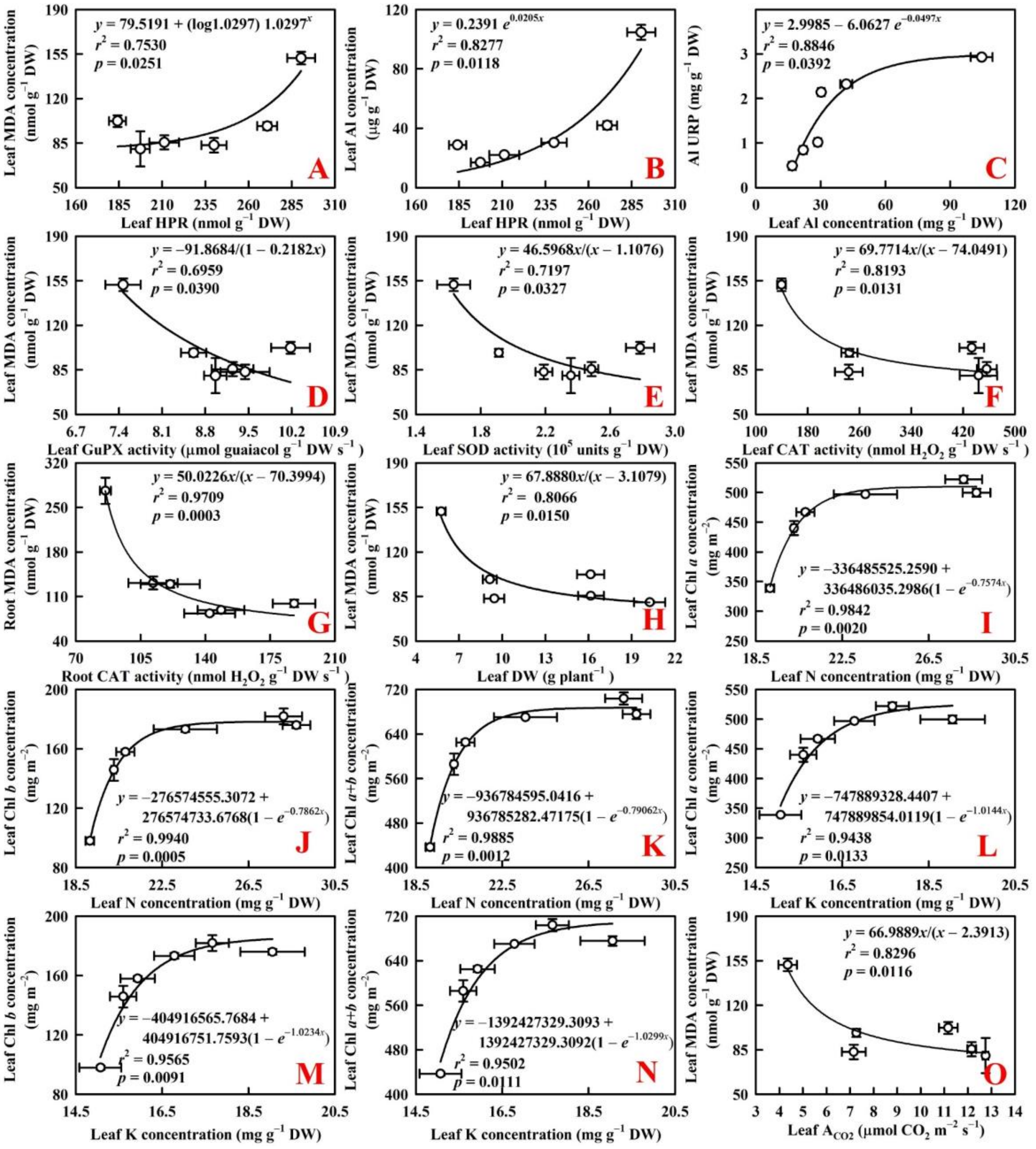

Figure S6. Curve regression analysis between some parameters. Each was the mean ± SE (n = 10 for leaf DW or 4 for other parameters) for the independent variables (horizontal) and the dependent variables (vertical).
