# Supplementary material for: Humic Acid Alleviates Aluminum Toxicity in Citrus grandis (L.) Osbeck: Insight from Growth, Gas Exchange, and Related Physiological Parameters
Source: Plants (Basel). 2026 Jul 31;15(15):2370. doi: 10.3390/plants15152370 (PMC13467780; doi:10.3390/plants15152370)
Supplement: Supplementary file 1 [file plants-15-02370-s001.zip › Table S2.pdf]

Table S2 Summary of parameters, formulae and their description using data extracted from chlorophyll (Chl) *a* (OJIP) transient

Fluoorescence parameters and calculation	***Description***
F_o	Minimum fluorescence, when all PSII reaction centers (RCs) are open
F_m	Maximum fluorescence, when all PSII RCs are closed
$F_v = F_m - F_o$	Maximum variable fluorescence
F_J	Fluorescence intensity at the J-step (2 ms)
F_I	Fluorescence intensity at the I-step (30 ms)
$\varphi_{Po} = TR_o/ABS = 1 - F_o/F_m = F_v/F_m$	Maximum quantum yield of primary photochemistry at $t = 0$
$M_o = 4\ (F_{300\mu s} - F_o)/(F_m - F_o)$	Approximated initial slope (in ms^{-1}) of the fluorescence transient V = f(t)
$F_v/F_o = (F_m - F_o)/F_o$	Maximum primary yield of photochemistry of photosystem II (PSII)
$V_I = (F_{30ms} - F_o)/(F_m - F_o)$	Relative variable fluorescence at the I-step (30 ms)
$V_J = (F_{2ms} - F_o)/(F_m - F_o)$	Relative variable fluorescence at the J-step (2 ms)
$ABS/RC = M_o/V_J/\varphi_{Po}$	Absorption flux per RC
$DI_o/RC = ABS/RC - TR_o/RC$	Dissipated energy flux per RC at $t = 0$
$ET_o/RC = M_o/V_J*\psi_o = M_o/V_J*(1-V_J)$	Electron transport flux per RC at $t = 0$
$RE_o/RC = (RE_o/ET_o)*(ET_o/RC)$	Reduction of end acceptors at PSI electron acceptor side per RC at $t = 0$
$\varphi_{Eo} = ET_o/ABS = F_v/F_m * (1 - V_J)$	Quantum yield for electron transport at $t = 0$
$\varphi_{Ro} = RE_o/ABS = \varphi_{Po}*\psi_{Eo}*\delta_{Ro}$	Quantum yield for the reduction of end acceptors of PSI per photon absorbed
$\psi_{Eo} = ET_o/TR_o = 1 - V_J$	Probability (at time 0) that a trapped exciton moves an electron into the electron transport chain beyond Q_A^-
$DI_o/CS_o = ABS/CS_o - TR_o/CS_o$	Dissipated energy flux per cross section (CS) at $t = 0$
$PI_{abs} = (RC/ABS)*(\varphi_{Po}/(1-\varphi_{Po}))*(\psi_{Eo}/(1-\psi_{Eo}))$	Performance index (PI) on absorption basis
$PI_{abs,total} = (RC/ABS)*(\varphi_{Po}/(1-\varphi_{Po}))*(\psi_{Eo}/(1-\psi_{Eo}))*(\delta_{Ro}/(1-\delta_{Ro}))$	Total performance index, measuring the performance up to the PSI end electron acceptors
